# Diet and monensin influence the temporal dynamics of the rumen microbiome in stocker and finishing cattle

**DOI:** 10.1186/s40104-023-00967-5

**Published:** 2024-01-26

**Authors:** Jianmin Chai, Caleb P. Weiss, Paul A. Beck, Wei Zhao, Ying Li, Jiangchao Zhao

**Affiliations:** 1 Guangdong Provincial Key Laboratory of Animal Molecular Design and Precise Breeding, College of Life Science and Engineering, Foshan University, Foshan, China; 2 Division of Agriculture, Department of Animal Science, University of Arkansas, Fayetteville, AR USA; 3 Department of Animal and Food Sciences, Oklahoma State University, Stillwater, OK USA; 4 Institute of Feed Research of Chinese Academy of Agricultural Sciences, Key Laboratory of Feed Biotechnology of the Ministry of Agriculture and Rural Affairs, Beijing, 100193 China

**Keywords:** Beef cattle, Diet, Feedlot phase, Monensin, Next-generation sequencing, Rumen microbiota, Stocker

## Abstract

**Background:**

Stocker cattle diet and management influence beef cattle performance during the finishing stage, but knowledge of the dynamics of the rumen microbiome associated with the host are lacking. A longitudinal study was conducted to determine how the feeding strategy from the stocker to the finishing stages of production affects the temporal dynamics of rumen microbiota. During the stocker phase, either dry hay or wheat pasture were provided, and three levels of monensin were administrated. All calves were then transported to a feedlot and received similar finishing diets with or without monensin. Rumen microbial samples were collected on d 0, 28, 85 during the stocker stage (S0, S28 and S85) and d 0, 14, 28, 56, 30 d before slaughter and the end of the trial during the finishing stage (F0, F14, F28, F56, Pre-Ba, and Final). The V4 region of the bacterial 16S rRNA gene of 263 rumen samples was sequenced.

**Results:**

Higher alpha diversity, including the number of observed bacterial features and the Shannon index, was observed in the stocker phase compared to the finishing phase. The bacterial amplicon sequence variants (ASVs) differentiating different sampling time points were identified. Dietary treatments during the stocker stage temporally impact the dynamics of rumen microbiota. For example, shared bacteria, including *Bacteroidales* (ASV19) and *Streptococcus infantarius* (ASV94), were significantly higher in hay rumen on S28, S85, and F0, while *Bacteroidaceae* (ASV11) and *Limivicinus* (ASV15) were more abundant in wheat. Monensin affected rumen microbial composition at a specific time. Transportation to feedlot significantly influenced microbiome structure and diversity in hay-fed calves. Bacterial taxa associated with body weight were classified, and core microbiotas interacted with each other during the trial.

**Conclusions:**

In summary, the temporal dynamics of the rumen microbiome in cattle at the stocker and finishing stage are influenced by multiple factors of the feeding strategy. Diet at the stocker phase may temporarily affect the microbial composition during this stage. Modulating the rumen microbiome in the steers at the stocker stage affects the microbial interactions and performance in the finishing stage.

**Supplementary Information:**

The online version contains supplementary material available at 10.1186/s40104-023-00967-5.

## Introduction

Ruminants, especially beef cattle, provide large amount of high-quality proteins for humans. The rumen, one of the most important and unique digestive organs for ruminants, has a complex microbial community that converts plant materials (i.e., cellulose and hemicellulose) mostly indigestible to humans to high-quality proteins through symbiotic microbiota fermentation [1, 2]. The function of the rumen microbiome is not only associated with nutrient utilization but also tightly related to host physiology and development, including the development of the rumen epithelium, possibly involving the modulation of host gene regulation by short-chain fatty acids [3–5]. Understanding of the rumen microbiome community has considerable benefits for livestock industry, as rumen dynamics are responsible for the ability of the host to obtain energy from the diet [6, 7]. In beef cattle, stocker and finishing operations are the two critical points of the production system. Stockers refer to weaned calves grazing pasture to enhance skeletal growth prior to finishing and slaughter. In other words, the growth and development in stocker steers significantly impact the performance of finishing cattle. Therefore, understanding the rumen microbiome of the stocker and finishing cattle is beneficial for feeding strategy and beef production.

Most recent studies solely focused on the factors influencing the rumen microbiome during the finishing stage [8–10]. However, there are many factors during the stocker and finishing stages could influence rumen microbiome and the host growth. A previous study found that the diversity and composition of rumen microbiome in Simmental crossbred cattle, yellow cattle, and cattle yak before and after transportation were significantly changed [11]. Another report found that a feed additive (monensin) affected the rumen fermentation of forage-fed beef cattle by modulating the rumen microbiome [12]. In addition, other factors, such as breed, sex, and host genetics, could also impact the rumen microbial community [13]. From the stocker to the finishing stage, calves experience complex processes of age and feeding strategy, including growth stage, dietary changes, transportation, and adding feed additives etc., which could significantly affect rumen microbiota and animal performance. However, many questions about factors influencing rumen microbiota in the stocker and finishing beef cattle remain unclear. For instance, how does the rumen microbiota change from the stocker to the finishing stage? What are the critical taxa in each stage and how do they change within one stage? Do early microbial colonizers affect late rumen bacterial composition? Are the microbial interactions associated with the diet or growth performance? To answer these questions, a comprehensive and longitudinal study of the rumen microbiome in beef cattle from the stocker to the finishing stage is needed.

In this study, to address these critical questions, the temporal dynamics in the rumen microbiome were characterized from the stocker to finishing stage. Signature microbiotas within and among stages were classified. It was also observed how the diet, feed additives, and transportation affected rumen microbial structure and identified the growth-associated taxa. Our results revealed the temporal dynamics of the rumen microbiota in beef cattle.

## Material and methods

All procedures were approved by the University of Arkansas Institutional Animal Care and Use Committee (Protocol #17018).

### Treatments and cattle

**Fig. 1:**
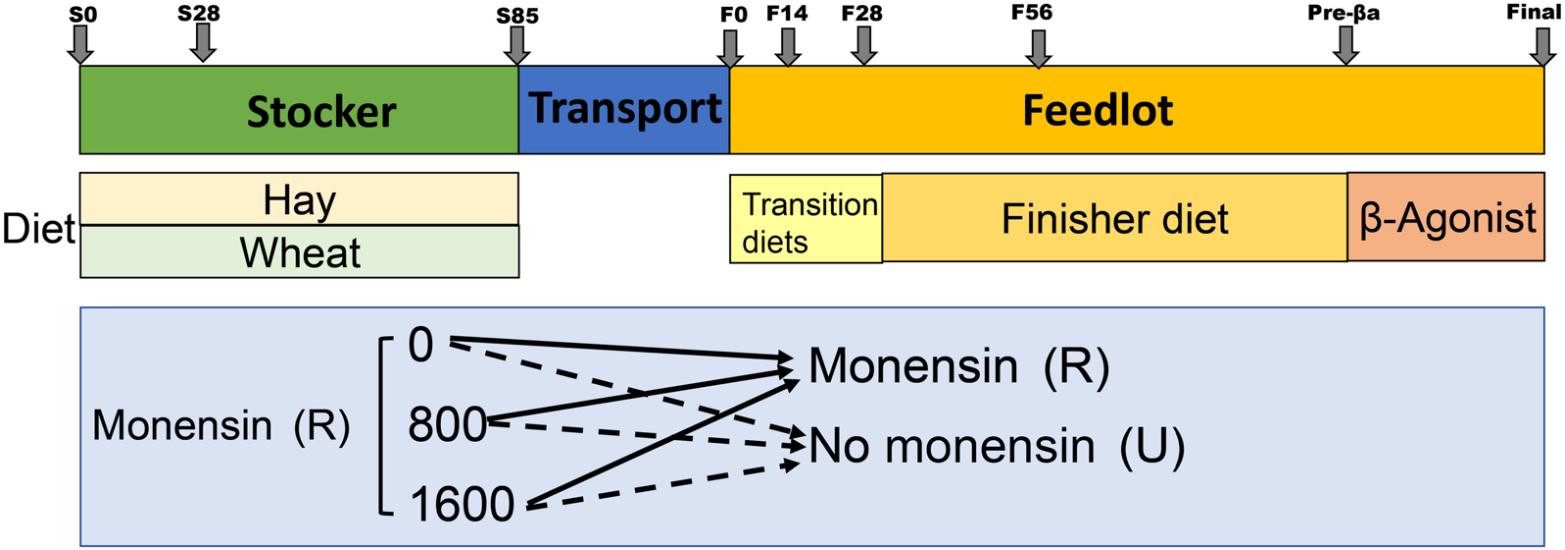
Experimental design and workflow. This study was conducted from the stocker to the finishing stage. Calves during the stocker stage were fed pearl millet (*Pennisetum glaucum*) hay with soybean hull and corn gluten feed supplement or grazed fall wheat (*Triticum aestivum*). Following the stocker phase, calves were transported 1,068 km to Canyon, TX, USA for finishing. At the feedlot, cattle were transitioned to a high-concentrate finisher diet for the first 28-d by replacing portions of corn stalks with steam-flaked corn weekly. Cattle remained on this diet for the remainder of the study until approximately 30-d prior to harvest, in which the beta-adrenergic agonists ractopamine hydrochloride (Pre-Ba) was included in the diet. Treatments for this experiment were arranged as a 3 × 2 factorial with factors consisting of three levels of monensin provided during the stocker phase, and whether or not monensin was provided in the feedlot diet following the stocker phase. Ruminal fluids were collected on d 0, 28, 85 (S0, S28, S85) during the stocker phase and on d 0, 14, 28, 56, starting to add Pre-Ba (30 d before harvest) and at the end of finishing stage (F0, F14, F28, F56, Pre-Ba, and Final) during the finishing stage

Crossbred beef steers [*n* = 167, body weight (BW) = 283 ± 27 kg] were obtained from the University of Arkansas Livestock and Forestry Research Station herd and were stratified by BW and assigned randomly to pasture and treatment. Calves were fed pearl millet (*Pennisetum glaucum*) hay with soybean hull and corn gluten feed supplement [0.5% BW daily (as-fed basis); Block 1] or grazed fall wheat (*Triticum aestivum*; Block 2) pasture (Fig. 1). Following the stocker phase, a subset of calves was transported 1,068 km to Canyon, TX, USA (blocks 1, 2) for finishing. Treatments for this experiment were arranged as a 3 × 2 factorial with factors consisting of the level of monensin provided during the stocker phase, and whether or not monensin was provided in the feedlot diet following the stocker phase. Treatments during the stocker phase consisted of offering grazing calves a free choice loose mineral (AMPT-A, ADM Animal Nutrition) with 0 g monensin/t (0R), 800 g monensin/t (800R), or 1,600 g monensin/t (1600R). Target daily mineral intake was 113 g/steer in order to provide monensin intakes of 0, 100, and 200 mg monensin/steer/d. When the stocker phase was completed, cattle were placed in a feedlot and the three previous treatments were split into two treatments in which monensin was provided at 0 mg/kg diet DM (U) or 37.5 mg/kg diet DM (M) in a mixed ration, for a total of 6 treatments for each block.

### Animal management

#### Block 1 of the stocker phase

On October 10 and 24, 2016, crossbred beef steers (*n* = 167) of similar age and body weight were obtained from the University of Arkansas Livestock and Forestry Research Station herd (*n* = 63) or were supplied by a local cooperator (*n* = 104) and transported to the University of Arkansas Livestock and Forestry Research Station in Batesville, AR. On December 7, 2016, approximately one-half of these calves (*n* = 84, BW = 302 ± 25 kg) were allocated to 1 of 12, 0.45 ha pastures (7 calves/pasture) with little residual forage mass and were allowed ad libitum access to large round bales of pearl millet hay (*Pennisetum glaucum*) fed in ring-style feeders and replaced as needed. Additionally, calves were supplemented daily with a soybean hull/corn gluten feed blend fed at 0.5% of BW (as-fed basis).

#### Block 2 of the stocker phase

The remaining steers (*n* = 83, BW = 260 ± 28 kg) not used in block 1 were allowed to graze wheat forage (*Triticum aestivum*) in 1 of 24 (3 or 4 calves/pasture) 1.6 ha dedicated wheat fields from December 7, 2016 to March 1, 2017. Wheat pastures were established using similar methods described by Beck at al. [14]. Briefly, the fields were prepared using one of two methods. Approximately one-half of the fields were prepared using a tilled method, in which fields were chisel plowed twice then disked twice with an offset disk and finished by disking an additional 2 times prior to planting to achieve a residual cover of < 5%. The remaining fields were prepared using a no-till method, in which they were prepared by applying 4.68 L/ha of glyphosate [*N*-(phosphonomethyl) glycine; Roundup Original Max, Monsanto Co., St. Louis, MO, USA] twice during the summer prior to planting to prevent residual plant growth. Wheat seed was then drilled into the previous crop residue. Pastures were planted using a grain drill in 17.8 cm rows at a depth of approximately 2.5 cm. Each pasture was fertilized in September with 68 kg of N/ha as ammonium nitrate.

#### Feedlot

After the stocker phase, steers were transported to the West Texas A&M Research Feedlot located near Canyon, TX for finishing. During initial processing, steers received an implant consisting of 200 mg trenbolone acetate and 40 mg of estradiol (Revalor-XS; Merck Animal Health, Madison, NJ, USA) and ivermectin (Noromectin, Norbrook Laboratories, Newry, UK). Cattle were blocked by pasture-off weights and allocated randomly, within previous stocker treatment to new treatment and pen (12 per set). Steers were housed in outdoor, uncovered 6.1 m × 26.9 m soil surface pens providing at least 23 m^2^ of pen space per steer. At the feedlot, cattle were transitioned to a high-concentrate finisher diet for the first 28 d by replacing portions of corn stalks with steam-flaked corn weekly. The final diet consisted of steam-flaked corn (37.3% of diet DM), corn gluten feed (43.5%), corn stalks (4.3%), corn oil (3.8%), molasses (7.3%), and a vitamin and mineral premix supplement (3.8%). Cattle remained on this diet for the remainder of the study until approximately 30-d prior to slaughter, in which the beta-adrenergic agonists ractopamine hydrochloride (Optaflexx, Elanco Animal Health, Greenfield, IN, USA) was included in the diet at 250 mg/head/d. Cattle were on feed for 132 and 164 d for the heavy and light blocks, respectively. Once cattle were visually determined to be market ready, cattle were transported to a commercial abattoir for slaughter.

### Sample collection

Ruminal fluids were collected on a subset of cattle (*n* = 30, 5 per treatment) from blocks 1 and 2 prior to the initiation of the stocker phase, on d 28, and at the end of the stocker phase before leaving the Batesville Station (Fig. 1). Samples were then collected approximately 12 h following the arrival at the feedlot in West Texas. Cattle did not have access to feed but did have ad libitum access to water between arriving at the feedlot and collecting the d 0 feedlot sample. Rumen microbial samples were also obtained 14, 28, and 56 d after arrival to the feedlot and immediately before a diet change to include ractopamine hydrochloride (Optaflexx, Pre-Ba, 101 and 131 DOF), approximately 30 d before slaughter. A final sample (Final) was collected prior to shipping cattle to a commercial abattoir for slaughter. The samples were obtained via the mouth using a sterilized stomach tube into 50-mL conical tubes and immediately placed in an insulated receptacle that contained dry ice and kept until transported to the laboratory. After arrival, samples were stored at −80 °C until analysis.

### DNA extraction, library preparation, and sequencing

The DNA from 263 rumen samples was isolated using a commercial microbial DNA isolation kit (DNeasy PowerSoils Kit, Qiagen Inc., Germantown, MD, USA) following the manufacturer’s protocol. Following isolation, DNA concentrations were determined using a spectrophotometer (Nanodrop One/C, Fisher Scientific, Hanover Park, IL, USA) and then diluted to 10 ng of DNA as required for library preparation. Samples were amplified by PCR using dual index primers (F515: 5′-GTGCCAGCMGCCGCGGTAA-3′ and R806: 5′-GGACTACHVGGGTWTCTAAT-3′), and selected to amplify the V4 region of the 16S rRNA gene in bacteria. Successful amplification was checked by using agarose gel electrophoresis and then the samples were normalized using a normalization kit (SequalPrep^TM^ Normalization Kit, Life Technologies, Grand Island, NY, USA). Following normalization, 5 μL aliquots from each sample were pooled to create a library. The library was sequenced using a next-generation sequencer (Illumina MiSeq® v2, San Diego, CA, USA) at the University of Arkansas Biomass Research Center in Fayetteville.

Raw sequencing files obtained from the Illumina sequencer were processed using the QIIME2 platform (2023.5 release) [15]. The demultiplexed sequences were processed using Deblur integrated with QIIME2 with default parameters [16], which included paired reads joining, length trimming, quality filtering, denoising (Deblur), classification (Greengenes2 reference database; 99% similarity), and sequence clustering [17]. Chimeric sequences and singletons were removed. Amplicon sequence variants (ASVs) were clustered based on 100% identity. All samples were rarefied to the minimum sample depth at 5,077 reads to reduce the effects of sequencing depth on alpha (Shannon index, observed ASVs) and beta (Bray-Curtis, Jaccard) diversity measures. Analysis of similarity (ANOSIM) was conducted to test the differences in beta diversity in QIIME2. The sequencing files of the current study are available in the Sequence Read Archive (SRA) repository (SUB12284212).

### Bioinformatics

Alpha diversity measures, including the Shannon index and the number of observed ASVs, were analyzed using the Wilcoxon Rank Sum Test in RStudio (RStudio Inc., Boston, MA, USA). Beta diversity based on the Bray-Curtis and Jaccard distance matrices was visualized on principal coordinate analysis (PCoA) plot. Differentially represented bacterial members between groups were determined using Galaxy LEfSe (https://huttenhower.sph.harvard.edu/lefse), with setting of LDA score over 3. An ASV table with metadata was imported into R-studio for further analysis and visualization. Regression-based Random Forest and the predict function in the R platform were used for the identification of the growth stage-associated bacterial ASVs. Network analysis of bacterial interactions was performed using the R package “psych”. The spearman correlations (*r*) were calculated to reveal the correlation between rumen bacteria at the ASV level with a correlation co-efficiency over 0.7 or less than −0.7, and then Cytoscape (version 3.8.2) software was used for visualization [18]. For all analyses, statistical significance was determined at *P* < 0.05.

## Results

### Temporal dynamics of the rumen microbiota from the stocker to the finishing phase

A total of 263 rumen samples were collected at nine-time points from the stocker to the finishing phase. A total of 4,769,452 high-quality reads from 9,114 ASVs at the single-nucleotide resolution were generated, with an average of 17,893 reads per sample. After the rarefaction of sample reads to 5,077, a total of 9,043 ASVs from 263 samples were included for downstream analysis of the rumen microbial community dynamics.

**Fig. 2:**
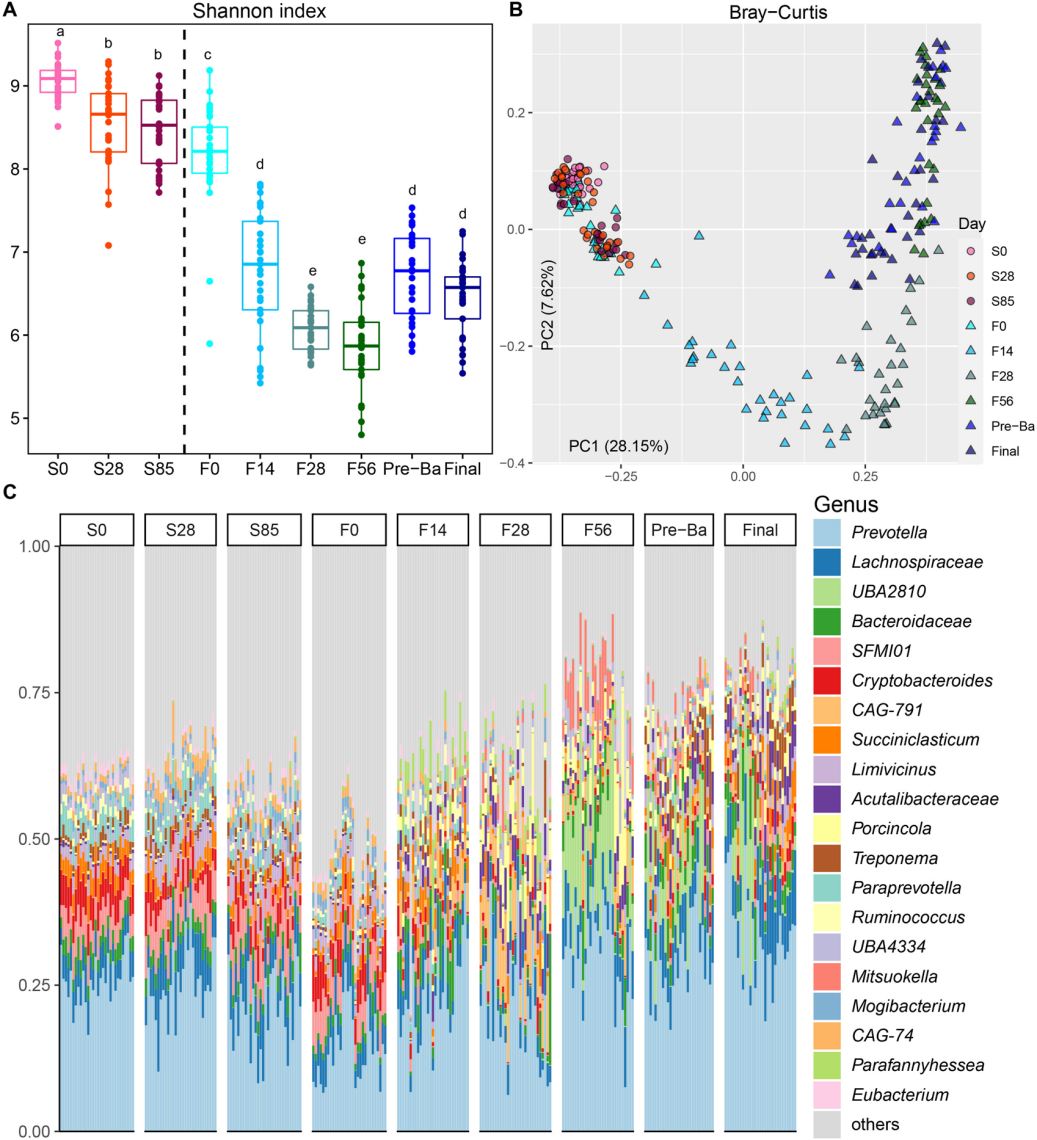
The temporal dynamics of rumen microbiota from the stocker to the finishing stage. **A** The Shannon index of rumen microbiota. The significance among sampling time points was labeled with different letters. **B** Principle Coordination Analysis (PCoA) of Bray-Curtis distances for microbiota. **C** Bacterial abundances at the genus level. Each bar represents a bacterial genus, and each column represents one sample. Ruminal fluids were collected on d 0, 28, 85 (S0, S28, S85) during the stocker phase and on d 0, 14, 28, 56, starting to add Pre-Ba (30 d before harvest) and at the end of finishing stage (F0, F14, F28, F56, Pre-Ba, and Final) during the finishing stage

Higher alpha diversity, including the number of observed ASVs and the Shannon index, was observed in the stocker phase compared to the finishing phase (Fig. 2A, Fig. S1A, Table S1). Specifically, the rumen microbiota at S0 had greater diversity than that at S28 and S85, and the Shannon index at S28 and S85 was higher compared to F0. After feedlot arrival and diet transition, alpha diversity of the rumen microbiome decreased significantly from F0 to F56, and increased on the day to add beta-adrenergic agonists ractopamine hydrochloride. However, after 1 month of consuming beta-adrenergic agonists ractopamine hydrochloride, the number of observed ASVs decreased. Significant shifts in community membership and structure from the stocker to the finishing phase were also observed on the PCoA plots based on Bray-Curtis (Fig. 2B, Table S2) and Jaccard (Fig. S1B, Table S3) distances. Samples on S0, S28, S85 and F0 clustered together, although they shaped two clusters compared to other sampling times. When receiving the transition diet at the early feedlot, F14 and F28 samples were distinct from those of the other three time points (F56, Pre-Ba and Final). With the amount of feedlot diet increasing in the transition diet, the rumen microbiome was more similar to F56, Pre-Ba and Final. Significant differences in beta diversity among F56, Pre-Ba and Final were also observed (Table S2 and S3).

At the phylum level, a total of 25 phyla, including Firmicutes, Bacteroidetes, Proteobacteria, and Actinobacteria, were observed across all samples, with Firmicutes being the most abundant phylum followed by Bacteroidetes accounting for 85% of the total sequences (Fig. S2). At the genus level, *Prevotella* were dominant genera throughout most of the stages (Fig. 2C). From S0 to the end of the finishing phase, the abundance of *Lachnospiraceae unclassified* and *Bacteroidaceae unclassified* kept consistent in the rumen. *SFMI01*, *Cryptobacteroides*, *Succiniclasticum* and *Limivicinus* were higher during the stocker phase and lower in the rumen of the finishing cattle. At the ASV level, the top 20 most abundant bacterial ASVs are displayed on stacked bar charts (Fig. S3). Among these top 20 taxa, significantly different ASV composition was observed between the stocker and finishing phase, and 9 belonged to *Prevotella* genus, the most dominant genus throughout the trial. *SFMI01* (ASV4), *Succiniclasticum ruminis* (ASV6) and *Prevotella* (ASV13) were abundant during the stocker phase, while *Succinivibrionaceae* *UBA2810* (ASV1) *Acutalibacteraceae* (ASV3), *Prevotella* (ASV5), and *Prevotella ruminicola* (ASV8) were enriched in the feedlot stage.

**Fig. 3:**
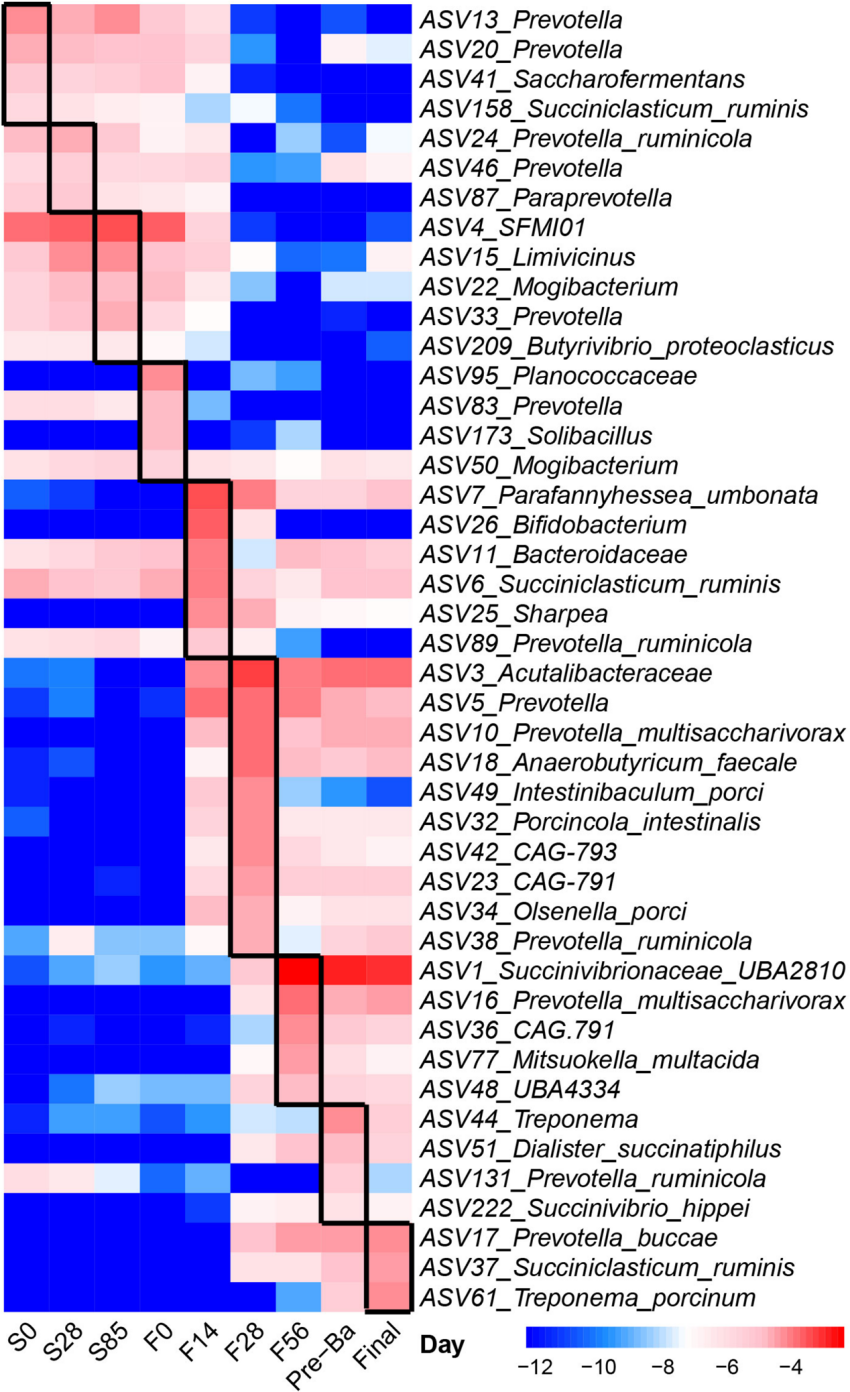
Microbial signatures for each sampling time point identified by LEfSe. A heatmap depicting the signature ASVs for each sampling time point identified by the LEfSe algorithm was drawn. The heat map shows the average relative abundances of ASVs on a log scale. The color of cells from blue to red corresponds to the relative abundance of ASVs from low to high. Ruminal fluids were collected on d 0, 28, 85 (S0, S28, S85) during the stocker phase and on d 0, 14, 28, 56, starting to add Pre-Ba (30 d before harvest) and at the end of finishing stage (F0, F14, F28, F56, Pre-Ba, and Final) during the finishing stage

The bacterial ASVs differentiating different sampling time points were identified using LEfSe, and the abundance of these ASVs is visualized on a heat map (Fig. 3). *Prevotella* (ASV13, ASV20), *Saccharofermentans* (ASV41) and *Succiniclasticum ruminis* (ASV158) were identified as the microbial signatures for S0 and had higher abundance through the stocker phase. The microbiota higher in S28, S85 and F0 had a similar pattern. For example, *Prevotella ruminicola* (ASV24) higher on S28, *SFMI01* (ASV4), *Limivicinus* (ASV15), *Mogibacterium* (ASV22) and *Prevotella* (ASV33) greater on S85, and *Prevotella* (ASV83) as the signature for F0 had higher abundance from S0 to F0, and then decreased at other ages of the finishing phase. *Solibacillus* (ASV173) and *Planococcaceae* (ASV95) were specifically higher on F0 and lower on other ages. Notably, *Mogibacterium* (ASV50) as microbial signature for F0 kept high abundance from S0 to the end of the trial. Similar pattern was also observed in *Bacteroidaceae* (ASV11) and *Succiniclasticum ruminis* (ASV6) that were abundant in F14. Other taxa, including *Parafannyhessea umbonata* (ASV7), *Bacteroidaceae* (ASV11) and *Sharpea* (ASV25), were enriched on F14 and continued to show high abundance until the end of the finishing phase. The bacterial signatures for F28 were *Acutalibacteraceae* (ASV3), *Prevotella* (ASV5, ASV10), *Anaerobutyricum faecale* (ASV18) and *Porcincola intestinalis* (ASV32) which had higher abundance from F14 to the end of the trial. *Succinivibrionaceae* *UBA2810* (ASV1), *Prevotella multisaccharivorax* (ASV16), *CAG-791* (ASV36) and *Mitsuokella multacida* (ASV77) were enriched on F56. *Treponema* (ASV44) was overrepresented on Pre-Ba. *Prevotella buccae* (ASV17), *Succiniclasticum ruminis* (ASV37) and *Treponema porcinum* (ASV61) were higher on Final.

### Dietary treatments during the stocker stage impacts the dynamics of rumen microbiota

**Fig. 4:**
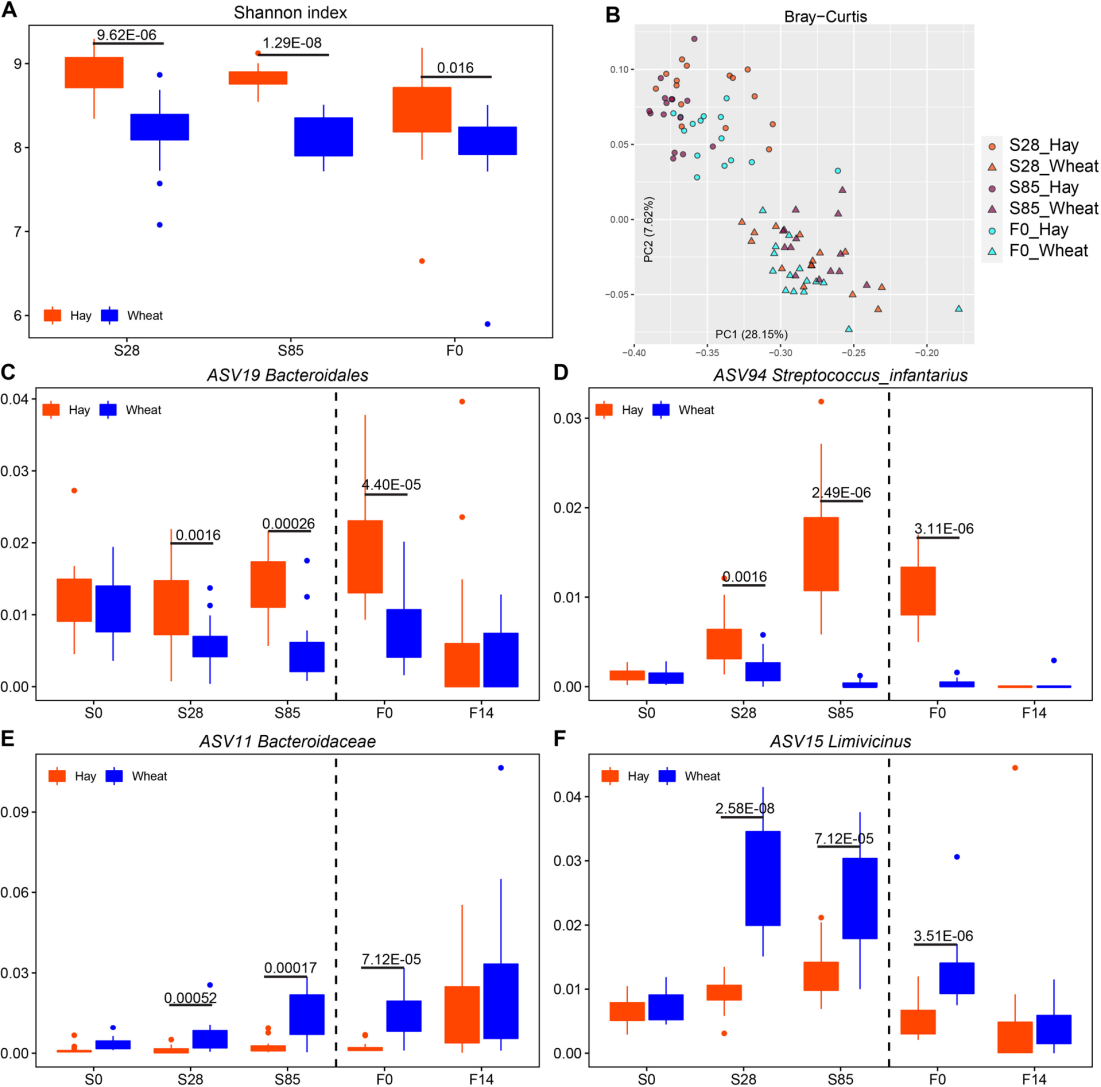
Diet affecting rumen microbial community. **A** The Shannon index of rumen microbiota on S28, S85, and F0 was affected by diet. The line inside the box denotes the median, and boxes denote the interquartile between the first and third quartiles (25^th^ and 75^th^ percentiles, respectively). The significances among sampling time points were labeled. **B** Principle coordination analysis (PCoA) of Bray-Curtis distances for microbiota on S28, S85, and F0. Each point represents one sample. **C**–**F** The boxplots of representative bacteria identified by LEfSe to display the microbial differences affected by diet. Cattle consumed different diet on d 0, 28, 85 (S0, S28, S85) during the stocker phase and on d 0 and 14, (F0 and F14) during the finishing stage

During the stocker phase, hay and wheat were the basal diet of two different blocks of steers; therefore, diet impacts on rumen microbiota from S0 to Final were analyzed. Alpha and beta diversity of the rumen microbiota on S28, S85 and F0 was mainly affected (Fig. S4). Both Shannon index and the number of observed ASVs were decreased in cattle grazing wheat compared to cattle fed hay (Fig. 4A, Fig. S5A). The microbial structure and membership on S28, S85 and F0 were more affected by a diet based on Bray-Curtis and Jaccard distance as samples in cattle consuming wheat were separated from the cattle receiving hay (Fig. 4B, Fig. S5B, Table S4 and S5). At the phylum and genus level, microbial differences by diet were also observed on S28, S85 and F0 (Fig. S6). For instance, Firmicutes phylum was higher in S28 wheat (48.39%) compared to S28 hay (39.89%), and *Prevotella* genus in wheat on S85 and F0 (23.85% and 18.42%) was significantly higher than that in hay at the same ages (18.22% and 10.38%). Next, LEfSe was performed to identify the bacterial ASVs differentiating hay and wheat on S28, S85 and F0 (Fig. S7). Shared ASVs among these three sampling time points were found. *Bacteroidales* (ASV19) and *Streptococcus infantarius* (ASV94) were significantly higher in hay rumen on S28, S85 and F0 (Fig. 4C and D), while *Bacteroidaceae* (ASV11) and *Limivicinus* (ASV15) were higher in wheat (Fig. 4E and F). Additionally, these four ASVs were not significantly different on S0 and F14.

### Monensin influences the rumen microbial community

**Fig. 5:**
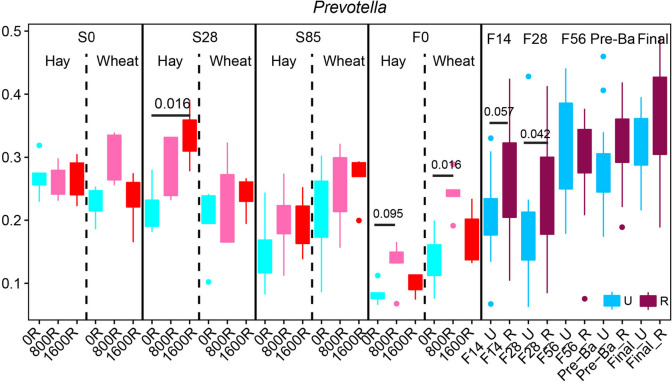
Monensin influencing the rumen microbial community. The dominant genus, *Prevotella*, was affected by adding monensin at a specific sampling time point. Significances were labeled at the time when they were statistically different. R = Monensin, U = No monensin. Three levels (0, 800, 1,600) of monensin during the stocker stage were designed. Ruminal fluids were collected on d 0, 28, 85 (S0, S28, S85) during the stocker phase and on d 0, 14, 28, 56, starting to add Pre-Ba (30 d before harvest) and at the end of finishing stage (F0, F14, F28, F56, Pre-Ba, and Final) during the finishing stage

The comparisons of three levels of monensin were determined under the effects of diet during the stocker phase to obtain accurate statistics for monensin impacts. No differences in Shannon index and the number of observed ASVs on S0, S85 and F0 were observed (Fig. S8). Notably, on S28, significant decreases in alpha diversity in 800R and 1600R groups were found compared to controls (0R), while the 800R and 1600R treatments were not different. The monensin effect during the finishing phase was also determined (Fig. S9). On F14, a significant decrease in Shannon index and the number of observed ASVs were found in R compared to U; however, higher alpha diversity in U was observed on F28. High observed ASVs were also found in R on F56, Pre-Ba and Final. No differences were found at other sampling time points. Likely, similar patterns were observed on the PCoA plot as significances were observed on S28 and F14 (Fig. S10 and S11). Moreover, the relative abundance of the dominant genus *Prevotella* was higher in monensin group on S28, F0 and F28 (Fig. 5). For instance, 1600R had a higher abundance of *Prevotella* than 0R on S28, and R on F28 was also higher. Although monensin increased its abundance in other ages, the statistic did not reach a significant level.

### Transportation affects rumen microbiota

**Fig. 6:**
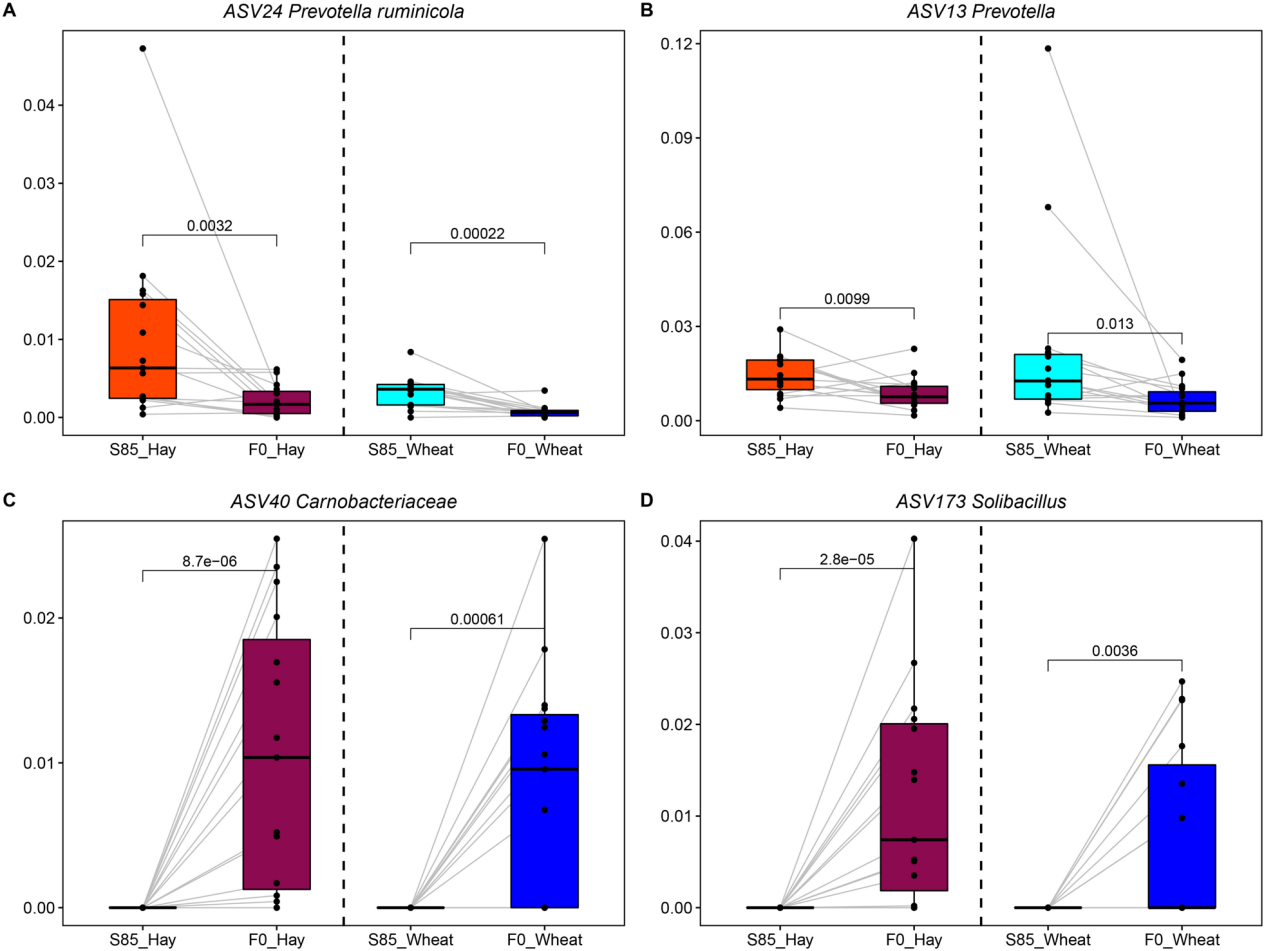
Transportation stress affected the rumen microbiota. **A**–**D** The boxplots of representative bacteria identified by LEfSe to display the microbial differences affected by transportation stress. The line inside the box denotes the median, and boxes denote the interquartile between the first and third quartiles (25^th^ and 75^th^ percentiles, respectively). The *P* values between groups were labeled

Transportation is a major stress during beef production. In this study, rumen microbiota associated with transportation was affected by diet. Interestingly, both the Shannon index and the number of observed ASVs in cattle consuming hay pasture were decreased after transportation (*P <* 0.05), while rumen microbial alpha diversity in cattle grazing wheat was not affected (Fig. S12 A and B). The structure and membership of rumen microbiota in both hay-fed and wheat-fed cattle were changed after transportation. Rumen microbial samples in hay-fed cattle on S85 were distinct compared to hay-fed cattle on F0 based on Bray-Curtis (ANOSIM: *r* = 0.59, *P =* 0.001) and Jaccard (ANOSIM: *r* = 0.63, *P =* 0.001). Rumen microbiota in wheat-fed cattle on S85 and F0 wheat clustered separately based on Bray-Curtis (ANOSIM: *r* = 0.31, *P =* 0.001) and Jaccard (ANOSIM: *r* = 0.43, *P =* 0.001) (Fig. S12 C and D). Next, signature microbiota for before- and after-transportation were identified by LEfSe. Shared ASVs were found, including *Prevotella ruminicola* (ASV24, ASV57), *Prevotella* (ASV13) and *Limivicinus* (ASV15), that were significantly decreased after transportation in both hay- and wheat-fed cattle (Fig. 6A and B, Fig. S13), while *Carnobacteriaceae* (ASV40), *Planococcaceae* (ASV135), *Psychrobacillus* (ASV170), *Solibacillus* (ASV173) and *Peribacillus psychrosaccharolyticus* (ASV308) were increased after transportation (Fig. 6C and D, Fig. S13). Other bacteria, including *Prevotella ruminicola* (ASV2, ASV89), *SFMI01* (ASV4), *Prevotella* (ASV20, ASV165), and *Mogibacterium* (ASV22), were decreased explicitly after transportation in the rumen from cattle grazing hay, while *Sacchaofermentans* (ASV41) was increased (Fig. S13A). Similarly, in the rumen community from cattle grazing wheat, *Prevotella* (ASV368 and ASV98) and *RF16* (ASV30) decreased after transportation, but *Succiniclastium ruminis* (ASV6, ASV58) increased (Fig. S13B).

### Growth performance-associated rumen microbiota

**Fig. 7:**
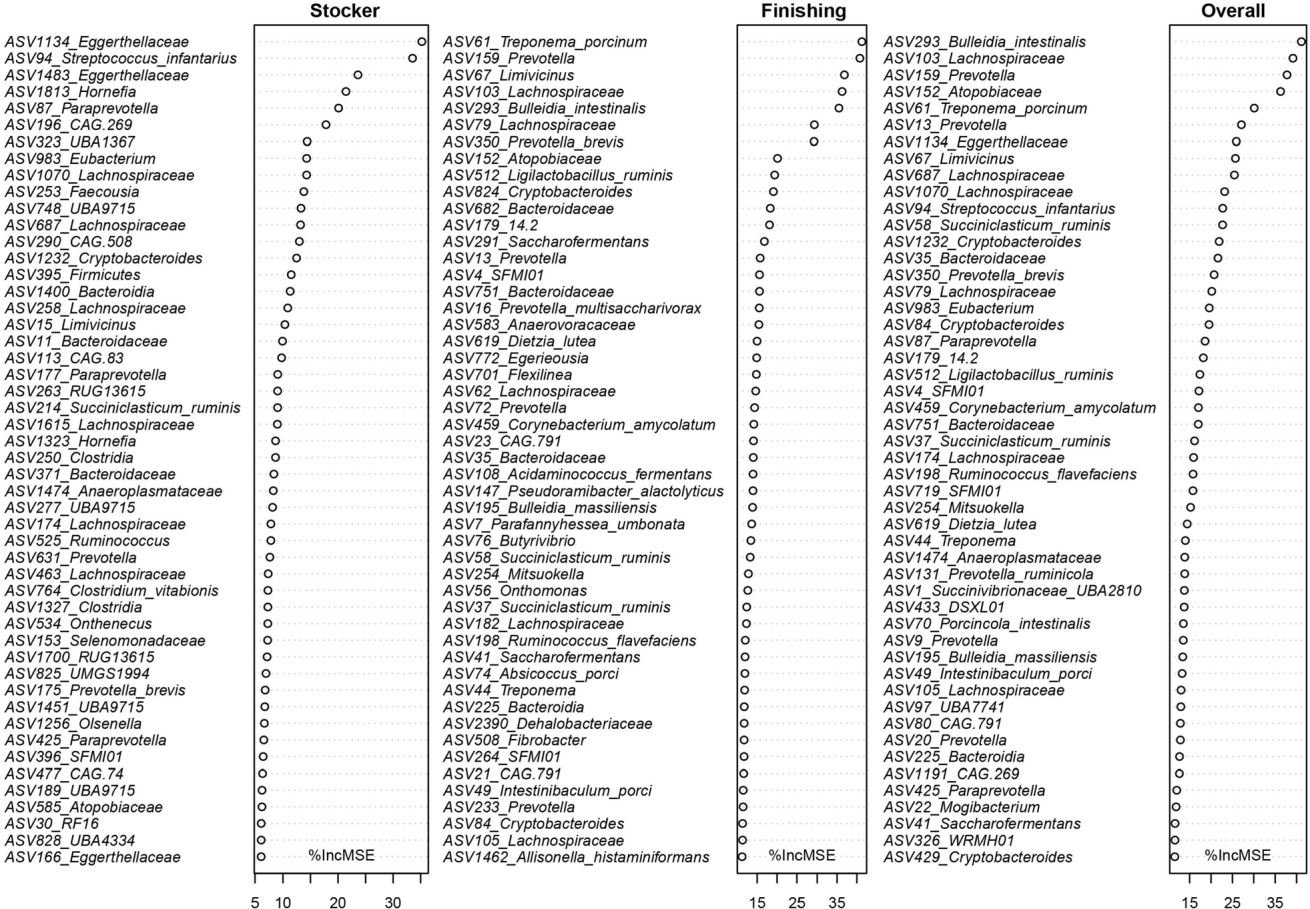
Growth performance related ASVs. The top 50 growth-related bacteria at the stocker, finishing, and overall stages were selected using regression-based random forest algorithm in R

To identify growth performance-associated bacterial taxa to be used as potential probiotics, regression-based random forest using BW as the outcome and the rumen bacterial ASVs as predictors were performed. Firstly, BW increased with growth stage (Fig. S14) and the diet effects were observed. The top 50 bacterial ASVs that predict growth performance are listed in Fig. 7. These predictors included members of stage-, diet- and transportation-associated ASVs. For example, *Limivicinus* (ASV15) and *RF16* (ASV30), were identified as signatures at the stocker phase, and *Parafannyhessea umbonata* (ASV7), *Bacteroidaceae* (ASV11), *Prevotella multisaccharivorax* (ASV16) and *Treponema porcinum* (ASV61) were listed as growth performance-related ASVs at the finishing stage. Moreover, rumen ASVs affected by diet and transportation were associated with growth performance, such as *Prevotella* (ASV13). In addition, the dominant taxa, such as *Succinivibrionaceae* *UBA2810* (ASV1), *SFMI01* (ASV4) and *Prevotella* (ASV9), were listed during the trial.

### Network analysis of the rumen microbial interactions in the stocker and finishing cattle

**Fig. 8:**
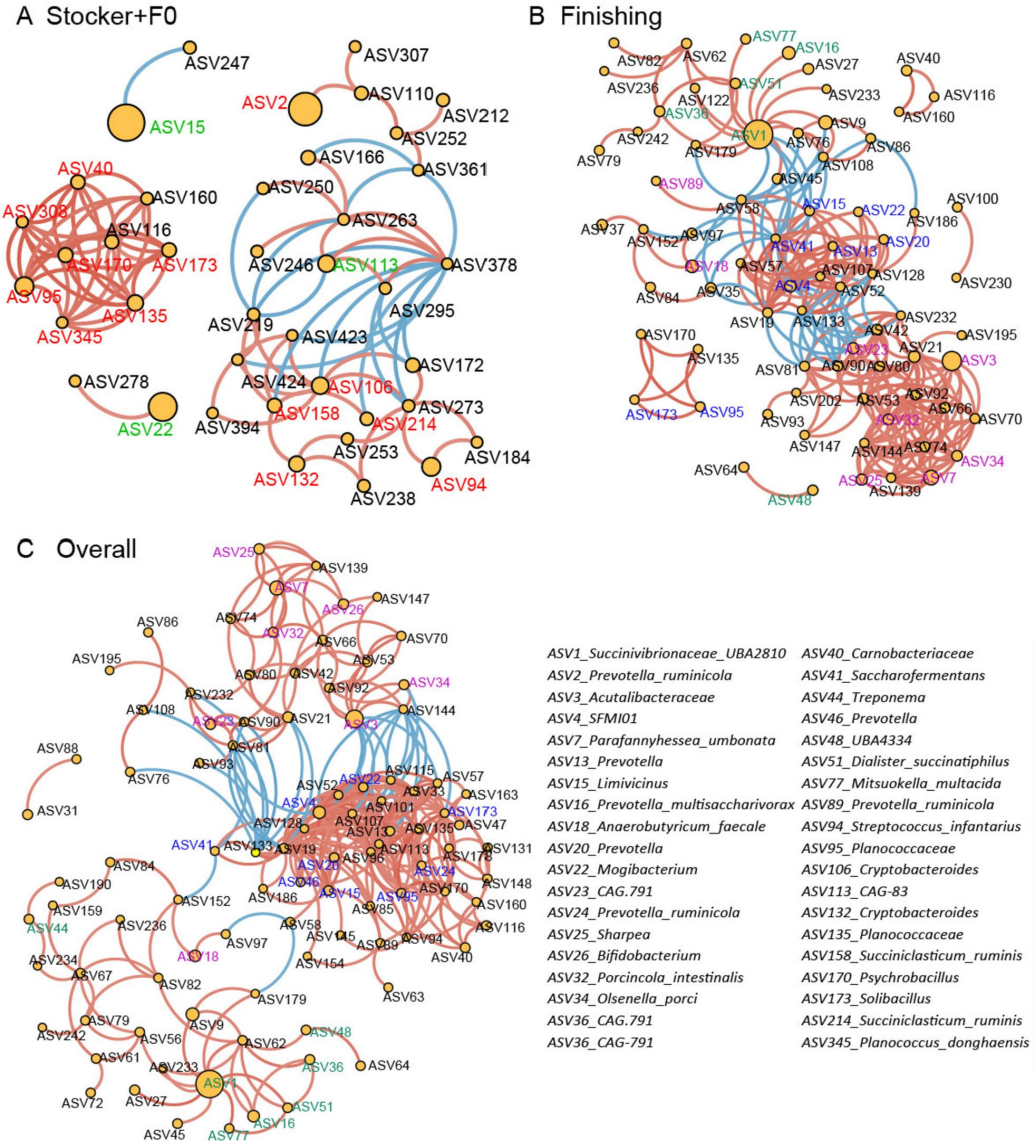
Network analysis of the interactions between bacterial taxa at different growth stages. Network analysis of bacterial interactions was performed using the R package “psych”. The spearman correlations (*r*) were calculated to reveal the correlation between rumen bacteria at the ASV level with a correlation co-efficiency over 0.7 or less than −0.7 and *P* < 0.05. Then, Cytoscape software was used for visualization. The yellow circles represent the relative abundance of each bacterial ASV. The blue lines mean a negative correlation between bacterial ASVs, while the red lines represent a positive correlation. F0: the timepoint to collect samples when cattle arrived feedlot

Rumen microbial interactions were determined using network analysis (Fig. 8). Firstly, using rumen samples from the stocker stage and F0, 5 modules with positive bacterial correlations within each module were observed (Fig. 8A). The bacterial ASVs identified for diet or transportation formed different clusters with negative correlations, indicating that diet (hay vs. wheat) effects and transportation stress affected microbial interactions. For example, *Limivicinus* (ASV15), *Mogibacterium* (ASV22) and *CAG-83* (ASV113) identified as signatures for wheat formed the key nodes within one module, while other modules included the hay signatures, such as *Streptococcus infantarius* (ASV94), *Carnobacteriaceae* (ASV40) and *Prevotella ruminicola* (ASV2). Next, microbial interactions during the finishing stage were estimated (Fig. 8B). The bacteria, including *Solibacillus* (ASV173), *Planococcaceae* (ASV95), *Limivicinus* (ASV15), *Mogibacterium* (ASV22), *SFMI01* (ASV4), and *Prevotella* (ASV13, ASV20), with lower abundance during the finishing stage positively interacted together. The microbial signatures for the diet transition period (F14 and F28), such as *Parafannyhessea umbonata* (ASV7), *Sharpea* (ASV25), *Acutalibacteraceae* (ASV3), *Porcincola intestinalis* (ASV32) and *Olsenella porci* (ASV34), shaped another module and positively correlated with each other. The microbiotas (i.e., *Succinivibrionaceae UBA2810* (ASV1), *Prevotella multisaccharivorax* (ASV16), *CAG-791* (ASV36) and *Mitsuokella multacida* (ASV77)) that were abundant on F56, Pre-Ba and Final had positive correlations and negatively correlated with other modules. Additionally, network analysis of all samples was performed (Fig. 8C). We found the signatures for each stage clustered. For example, ASV4, ASV20, ASV22, ASV15, ASV24, ASV41 and ASV95 as dominant ASVs in the rumen during the stocker stage gathered, while ASVs with high abundance in the finishing stage rumen positively correlated with each other. Moreover, a negative correlation between the two modules was observed.

## Discussion

Finishing cattle are a critical meat source for humans. Understanding the rumen microbiome in cattle from a stocker to finishing stage is beneficial for feeding strategy and beef production as they play a vital role in the breakdown and utilization of feedstuff carbohydrates and protein [7, 19]. This study characterized the temporal dynamics of the rumen microbiome from the stocker to finishing stage that experienced changes of age, diet, monensin addition and transportation. We identified the signature bacteria for each sampling time point. The effects of diet, monensin and transportation on the rumen microbiome were investigated and confirmed these factors influencing the microbial community. The growth-associated taxa were also classified by a machine learning algorithm. The microbial interactions were associated with all these factors, which provides insights into the mechanism that feeding strategy manipulates the rumen microbiota and improves the growth performance of beef cattle. This study revealed the mechanism of how feeding strategy influenced microbiome dynamics.

### Temporal dynamics of the rumen microbiota in beef cattle from the stocker to finishing stages are associated with the key taxa

Investigation of temporal dynamics of rumen microbiota allowed us to understand the microbial changes from the stocker to the finishing stage. Then, the microbial changes associated with the growth stage and dietary changes could be found, which provide insights into feeding management [20, 21]. Significant differences in rumen microbial communities between the stockers and finishing cattle were found based on alpha and beta diversities, indicating the changes in the microbiome that are linked to age and diet. The stocker calves were stocked on available forage (wheat or hay), while a high-concentrate ration at a feedlot in a stepwise manner was provided for the finishing cattle, which is a classical feeding model in the southern US [22]. As the dominant genus, *Prevotella* showed higher relative abundance in the finishing stage compared to the stocker stage in response to alterations from a fiber-based diet to a high-concentrate diet, which was confirmed by Ramos et al. [23]. *Prevotella*, a well-known degrader of starch, β glycans, protein, pectin, and hemicellulose, allows its ability to dominate in the rumen under a range of diets since it can use a variety of substrates [24]. This study found that different ASVs associated with *Prevotella* were higher in either the stocker or finishing stage. It reflects that the species or sub-species under *Prevotella* had different responses to fiber- or high-concentrate diets. In addition, *Succinivibrionaceae* family increased significantly from the stocker to finishing stage and *Cryptobacteroides* family decreased, which was reported as diet-associated bacterial changes [25]. Correspondingly, the microbial interactions were also in accordance with the growth stage. Therefore, the temporal dynamics of rumen microbiome are associated with the animal development and diet.

### Diet in the stocker stage affects the rumen microbiome in feedlot cattle

Microbial species affected by diet at early life stages have a long-term impact on in rumen ecosystem [26]. In this study, the effects of dietary treatments during the stocker stage on the assembly dynamics of rumen microbiota were estimated. The microbial diversity and structure were affected from the stocker stage to the first day at the feedlot. Similarly, increases of *Bacteroidales* (ASV19) and *Streptococcus infantarius* (ASV93) by hay and *Bacteroidaceae* (ASV11) and *Limivicinus* (ASV15) by wheat had the same pattern. Previous studies reported that a corn stover-based diet increased the relative abundance of *Streptococcus* [27] and particular species of the *Bacteroidaceae* family to be more enriched in sheep received a concentrate diet compared to those fed a forage-based diet, which is in accord with these results. This may be related to cellulolytic or non-cellulolytic ferment from hay or wheat [28]. When cattle started to consume the finishing diet, the impacts of the stocker diet on rumen microbiota declined. This phenomenon was also described and confirmed by Rey et al. [29] and Furman et al. [25], where an increase in a specific genus was detected during a period of a specific diet consumption. The importance of these ASVs associated with growth performance was confirmed with a machine learning technique, a regression based Random Forest, that use the relative abundance of these ASVs as predictors, indicating that they play critical roles in nutrient digestion and metabolism. In addition, these ASVs interacting with other bacteria in the finishing rumen community was found. It further explains that diet framing the rumen microbiota influences the subsequent rumen ecosystem. Further experiments are needed to culture these bacteria and test their functions in animal performance and rumen microbiota development.

### Monensin supplementation influences the rumen microbiome in cattle

Monensin is a class of carboxylic polyether ionophore antibiotics which have the ability to modify rumen fermentation and increase the body weight of cattle [21]. However, its effects on the rumen microbiome have not been fully described. In the current study, monensin decreased the diversity of the rumen microbiome in a short time. Scharen et al. [30] also found monensin decreased rumen bacterial diversity in postpartum cows. In detail, the relative abundance of *Prevotella* genus was increased by monensin at a specific sampling timepoint, which has the same results of which monensin increased the relative abundance of *Prevotella dentalis* and *Prevotella brevis* in forage-fed beef cattle [12]. The impacts of monensin on rumen microbiota are controversial. A previous report did not find significant changes in rumen microbiota in heifers receiving 70 d monensin [31]. Another study found that with this diet, the medium dosage (368 mg/cow/d) of monensin was most efficacious for the changes of bacteria in the rumen of lactating dairy [32]. Therefore, the effects of monensin on the ruminal microbiome might be time-dependent as no differences were observed in late sampling time points. The rumen microbial ecosystem was reshaped through a series of succession processes after the adaption to monensin [33]. These results are supported by findings by Gadberry et al. [34] in a meta-analysis where increased study duration had a negative influence on ADG response to monensin, indicating an increasing tolerance of rumen microbes to monensin over time. Further studies need to deeply investigate the microbiome changes in response to monensin.

### Transportation stress impacting the rumen microbiome in cattle might be associated with diet

Transportation to feedlot is a normal practice in beef cattle production. Due to transportation stress and feed and water restriction, the nutrition-metabolism balance, hormone secretion levels, and immune competence can result in negative effects on cattle physiology, diseases, and growth [11]. For researches related to the US beef cattle, most studies focus on the bovine respiratory disease caused by shipping [35]. Although transportation stress has been reported to affect rumen cellulolytic microbiota, such as *Prevotella ruminicola*, studies investigating the effects of transportation on rumen microbiota in cattle receiving different diets are lacking [36, 37]. This study found that the Shannon index of the rumen microbiota in hay-fed cattle changed significantly after transportation, while wheat-fed cattle did not show changes in rumen microbial alpha diversities. It is possible that transportation mainly affects cellulolytic bacteria that are dominant in rumen receiving hay diet. On the other side, wheat with high non-fiber carbohydrates might be another factor to resist transportation stress [38]. Moreover, wheat effects on the rumen microbiome were observed from d 28 of the stocker phase, which may affect the core microbiota and stress. Although the mechanisms of no changes in the diversity of the rumen community in transported cattle cannot be elucidated with these data, intake of wheat could decrease the side effects of transportation on the rumen microbiome in the beef cattle. Additionally, we found that consistently changed ASVs in both hay and wheat diet group. *Prevotella ruminicola* (ASV24, ASV57), and *Prevotella* (ASV13) were significantly decreased after transportation in both hay and wheat diet groups, which is the same as a previous study [37]. ASVs of *Solibacillus* in the rumen increased with transportation. Genus *Solibacillus* in the ruminants’ gut has been associated with a reduction in diet intake, possibly as an adaptive response to increased dietary variability during the transportation [39, 40]. In addition, a higher abundance of *Solibacillus* in the nasopharyngeal microbiome of calves with the bovine respiratory disease was reported [41]. This suggests that transportation stress results in decreases in resident bacteria and increases in bacteria associated with pathogens in the rumen; however, its negative impacts are related to the type of diet consumed. Additionally, increases in rumen pathogens after transportation that may disperse to the bovine respiratory tract for disease causes are needed to be investigated in the future studies. Of note, these changes in the rumen microbiota could be attributed to transportation itself and also other factors such as fasting and new environment. Future studies are desired to distinguish the contributions of each factor to such changes in rumen microbiota during transportation.

### The rumen microbiome in cattle is associated with growth performance

The fermentation products from the rumen microbiota contribute to the host’s nutrient supply and modulation of the rumen microbiome to improve performance has become a novel and effective strategy in the livestock industry [5]. In the current study, the top rumen bacterial taxa that are most related to BW were identified by a random forest model. The bacteria associated with diet and stages were correlated with BW, indicating their roles in nutrient digestion and host growth. Moreover, they interacted with each other based on the network analysis. For instance, *Succinivibrionaceae* (ASV1), the predominant taxon in the rumen of finishing cattle, was associated with BW and was the key node in the network analysis. Of note, *Succinivibrionaceae* family identified as core rumen microbiota is associated with carbohydrate metabolism and nitrogen utilization [42]. It was reported as succinate and acetate producers via carbohydrate fermentation [43–46]. A study reported that microbial composition, and particularly, the specific characteristics of microbe-microbe interactions, are also correlated with body weight gain [47]. Hence, core microbiota in rumen might interact with other bacterial taxa, producing volatile fatty acids and consequently promoting growth performance.

## Conclusions

In summary, this research systematically characterized the temporal dynamics of rumen microbiome in cattle at the stocker and finishing stage influenced by multiple factors of feeding strategy. Diet during the stocker stage may temporarily affect the microbial composition during this stage, and monensin supplementation could affect the microbial structure in a short period. Although wheat diet feeding could decrease the side effects of transportation stress on the rumen microbial community to some extent, decreases in dominant bacteria and increases in pathogenic microbiota are found regardless of diet consumption. Moreover, the rumen microbiome and its interactions are associated with growth performance in beef cattle at the stocker and finishing stages. These results implicate that producers could use the optimal feeding strategy to modulate the rumen microbiome of their herd and program the subsequent production.

### Supplementary Information

**Additional file 1: Fig. S1.** Alpha and beta diversity of the temporal dynamics of the rumen microbiome. **Fig. S2.** Temporal dynamics of the microbial composition at the phylum level. **Fig. S3.** Temporal dynamics of the top 20 ASVs in rumen. **Fig. S4.** Dietary treatment during the stocker stage influences on the rumen microbiota during the whole trial. **Fig. S5.** Dietary treatments during the stocker stage impact the rumen microbiota during the whole trial. **Fig. S6.** Dietary treatments during the stocker stage impact the rumen microbiota at the phylum and genus level during the whole trial. **Fig. S7.** Rumen microbiota signatures for diet at the ASV level during the stocker stage. **Fig. S8.** Effect of diet and monensin level on the alpha diversity in the rumen during the stocker phase. **Fig. S9.** Effect of monensin on the alpha diversity in the rumen during the finishing phase. **Fig. S10.** Effect of diet and monensin on beta diversity in the rumen during the stocker phase. **Fig. S11.** Effect of monensin on beta diversity in the rumen during the finishing phase. **Fig. S12.** Beta diversity in the rumen of cattle consuming hay and wheat diet before and after transportation. **Fig. S13.** Transportation associated bacteria identified by LEfSe. **Fig. S14.** Body weight of cattle from the stocker to finishing stage. **Table S1.** Differences in alpha diversities of the rumen microbiome at different growth stages. **Table S2.** Dissimilarities in the rumen microbiome at different growth stages revealed by analysis of similarity (ANOSIM) based on Bray-Curtis distances. **Table S3.** Dissimilarities in the rumen microbiome at different growth stages revealed by analysis of similarity (ANOSIM) based on Jaccard distances. **Table S4.** Dissimilarities in the rumen microbiome of cattle consuming different diets revealed by analysis of similarity (ANOSIM) based on Bray-Curtis distance. **Table S5.** Dissimilarities in the rumen microbiome of cattle consuming different diets revealed by analysis of similarity (ANOSIM) based on Jaccard distance.

## Abbreviations

ADG: Average daily gain
ANOSIM: Analysis of similarity
ASVs: Amplicon sequence variants
BW: Body weight
DM: Dry matter
PCoA: Principal coordinate analysis
Pre-Ba: Time to add beta-adrenergic agonists ractopamine hydrochloride
SRA: Sequence read archive
