# Supplementary material for: Diet and monensin influence the temporal dynamics of the rumen microbiome in stocker and finishing cattle
Source: J Anim Sci Biotechnol. 2024 Jan 26;15:12. doi: 10.1186/s40104-023-00967-5 (PMC10811932; doi:10.1186/s40104-023-00967-5)
Supplement: Supplementary file 1 — Additional file 1: Fig. S1. Alpha and beta diversity of the temporal dynamics of the rumen microbiome. Fig. S2. Temporal dynamics of the microbial composition at the phylum level. Fig. S3. Temporal dynamics of the top 20 ASVs in rumen. Fig. S4. Dietary treatment during the stocker stage influences on the rumen microbiota during the whole trial. Fig. S5. Dietary treatments during the stocker stage impact the rumen microbiota during the whole trial. Fig. S6. Dietary treatments during the stocker stage impact the rumen microbiota at the phylum and genus level during the whole trial. Fig. S7. Rumen microbiota signatures for diet at the ASV level during the stocker stage. Fig. S8. Effect of diet and monensin level on the alpha diversity in the rumen during the stocker phase. Fig. S9. Effect of monensin on the alpha diversity in the rumen during the finishing phase. Fig. S10. Effect of diet and monensin on beta diversity in the rumen during the stocker phase. Fig. S11. Effect of monensin on beta diversity in the rumen during the finishing phase. Fig. S12. Beta diversity in the rumen of cattle consuming hay and wheat diet before and after transportation. Fig. S13. Transportation associated bacteria identified by LEfSe. Fig. S14. Body weight of cattle from the stocker to finishing stage. Table S1. Differences in alpha diversities of the rumen microbiome at different growth stages. Table S2. Dissimilarities in the rumen microbiome at different growth stages revealed by analysis of similarity (ANOSIM) based on Bray-Curtis distances. Table S3. Dissimilarities in the rumen microbiome at different growth stages revealed by analysis of similarity (ANOSIM) based on Jaccard distances. Table S4. Dissimilarities in the rumen microbiome of cattle consuming different diets revealed by analysis of similarity (ANOSIM) based on Bray-Curtis distance. Table S5. Dissimilarities in the rumen microbiome of cattle consuming different diets revealed by analysis of similarity (ANOSIM) based on Ja [file 40104_2023_967_MOESM1_ESM.docx]

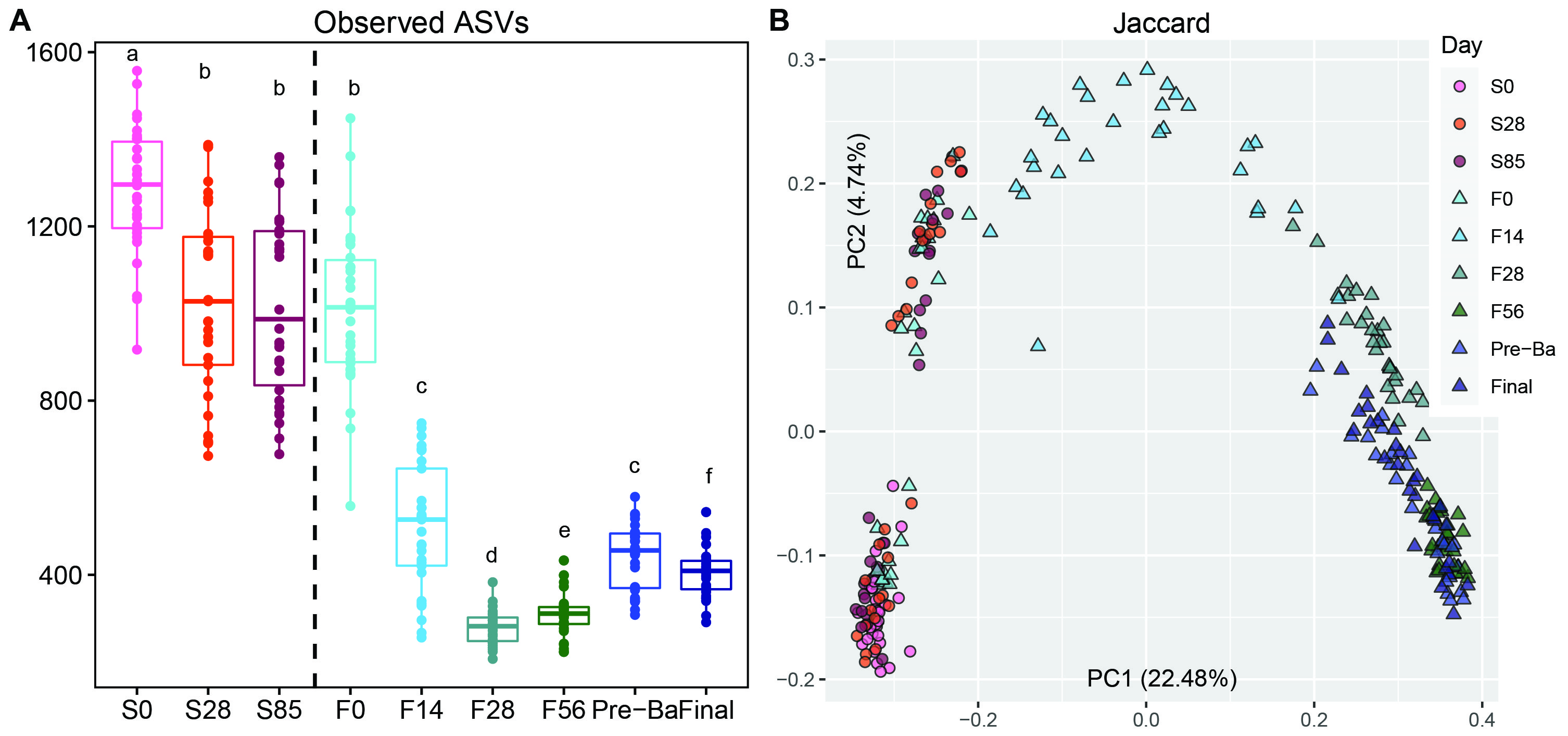


**Fig. S1** Alpha and beta diversity of the temporal dynamics of the rumen microbiome


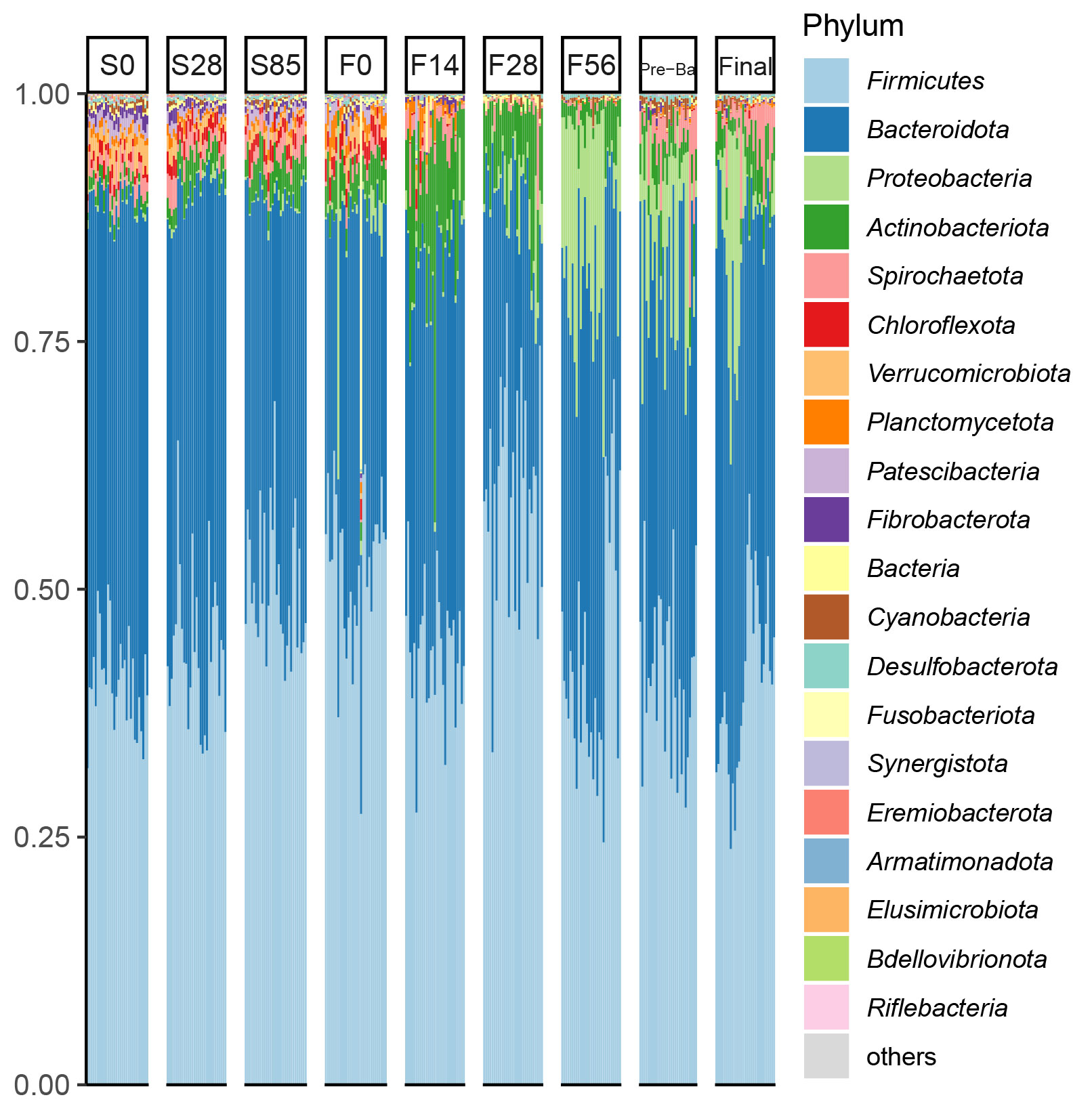


**Fig. S2** Temporal dynamics of the microbial composition at the phylum level


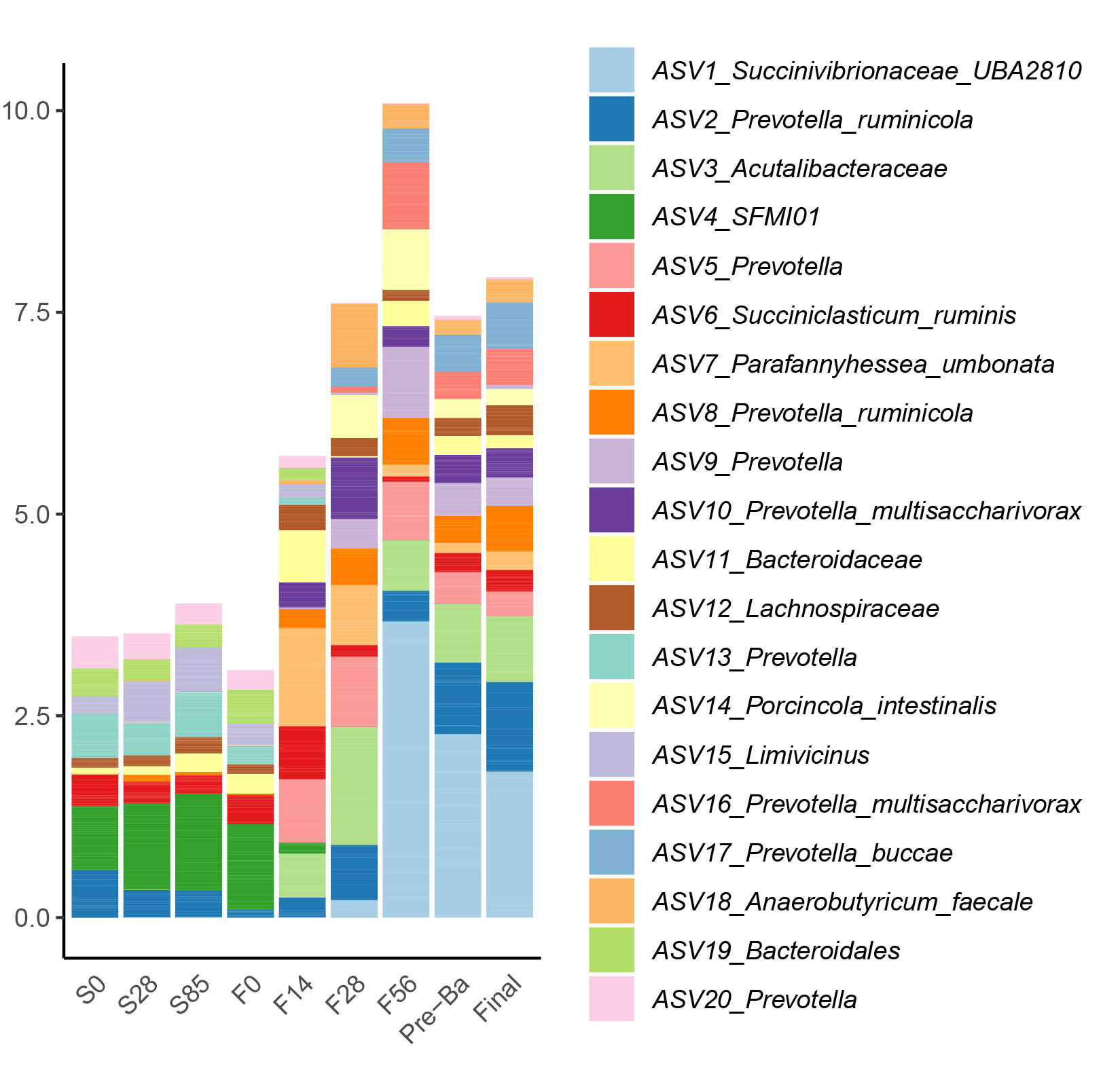


**Fig. S3** Temporal dynamics of the top 20 ASVs in rumen


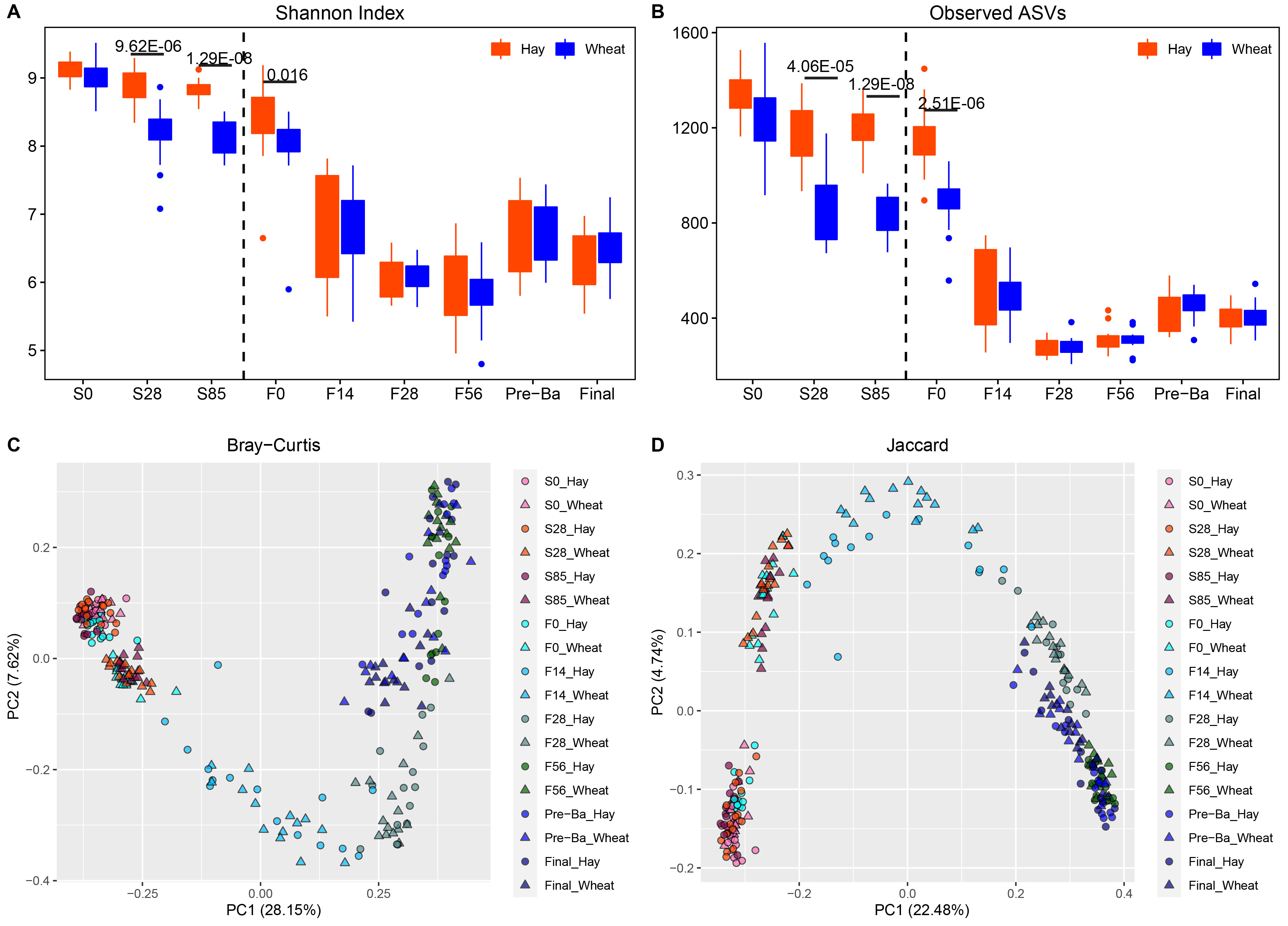


**Fig. S4** Dietary treatment during the stocker stage influences on the rumen microbiota during the whole trial


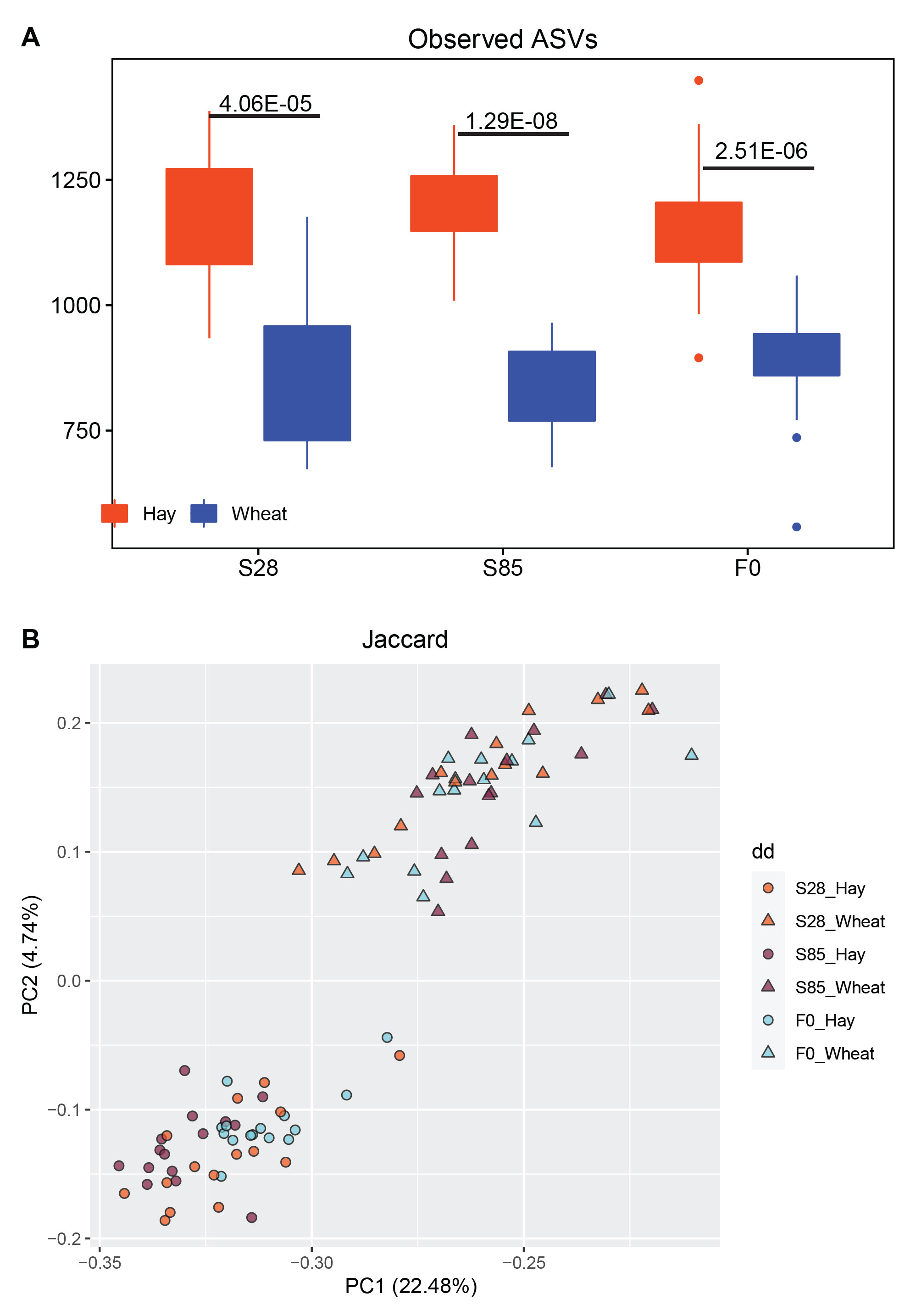


**Fig. S5** Dietary treatments during the stocker stage impact the rumen microbiota during the whole trial


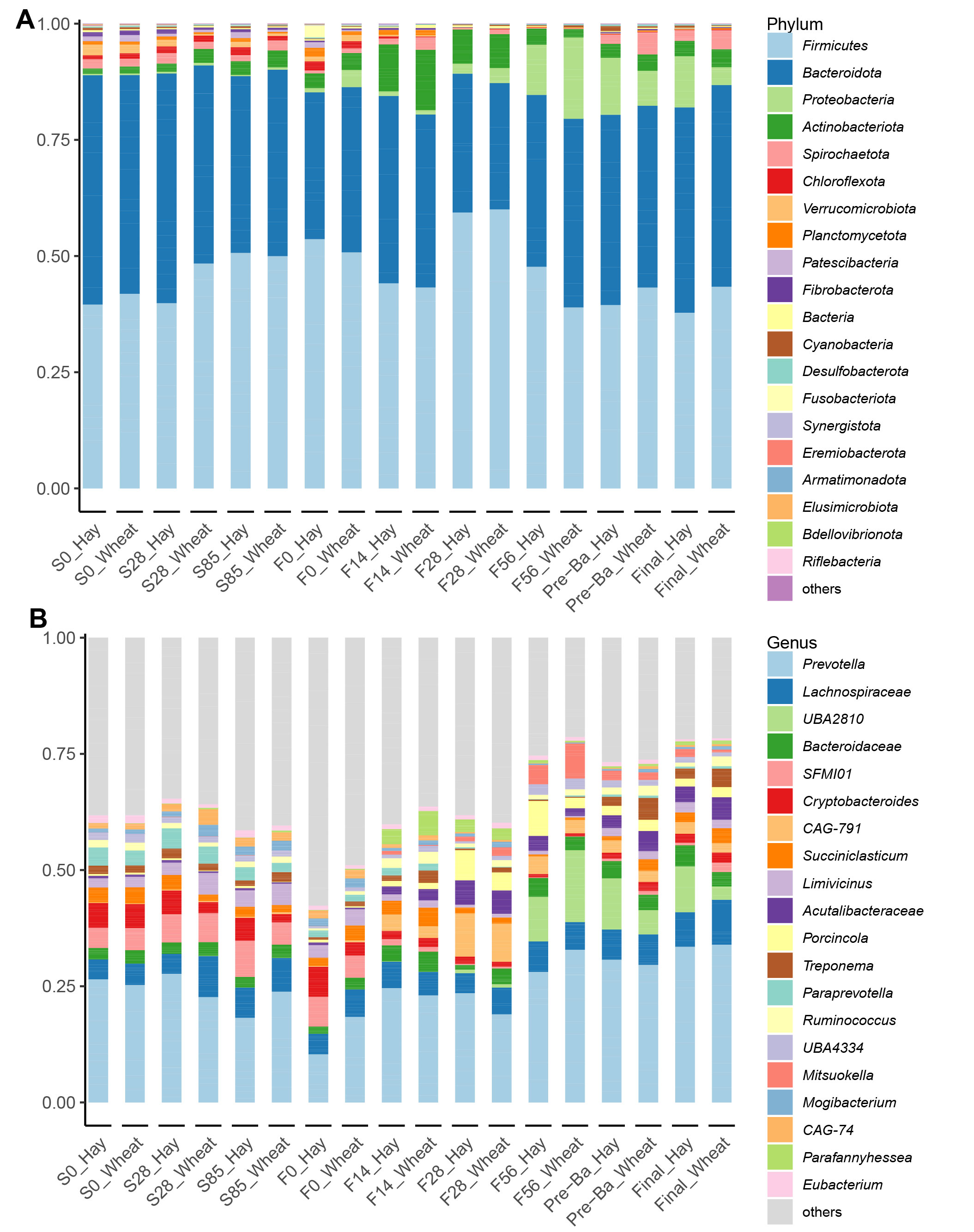


**Fig. S6** Dietary treatments during the stocker stage impact the rumen microbiota at the phylum and genus level during the whole trial


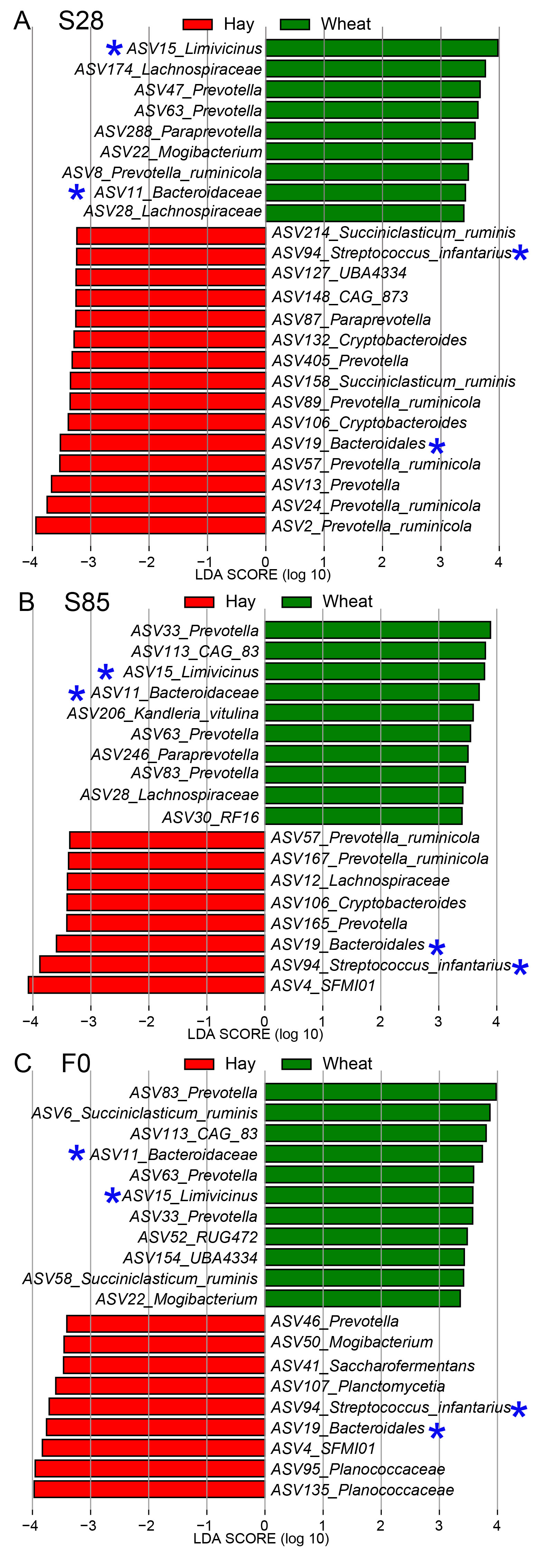


**Fig. S7** Rumen microbiota signatures for diet at the ASV level during the stocker stage


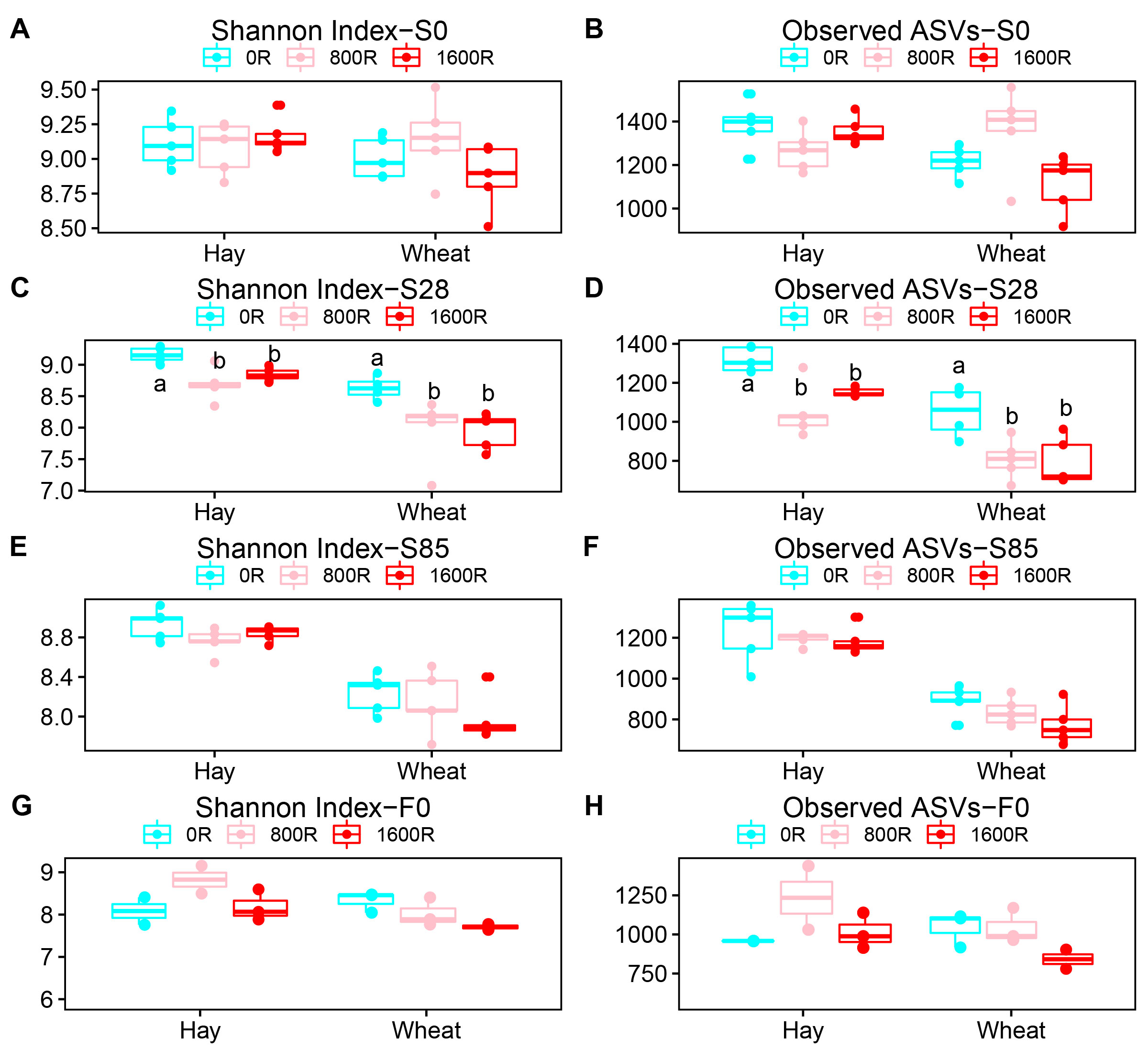


**Fig. S8** Effect of diet and monensin level on the alpha diversity in the rumen during the stocker phase


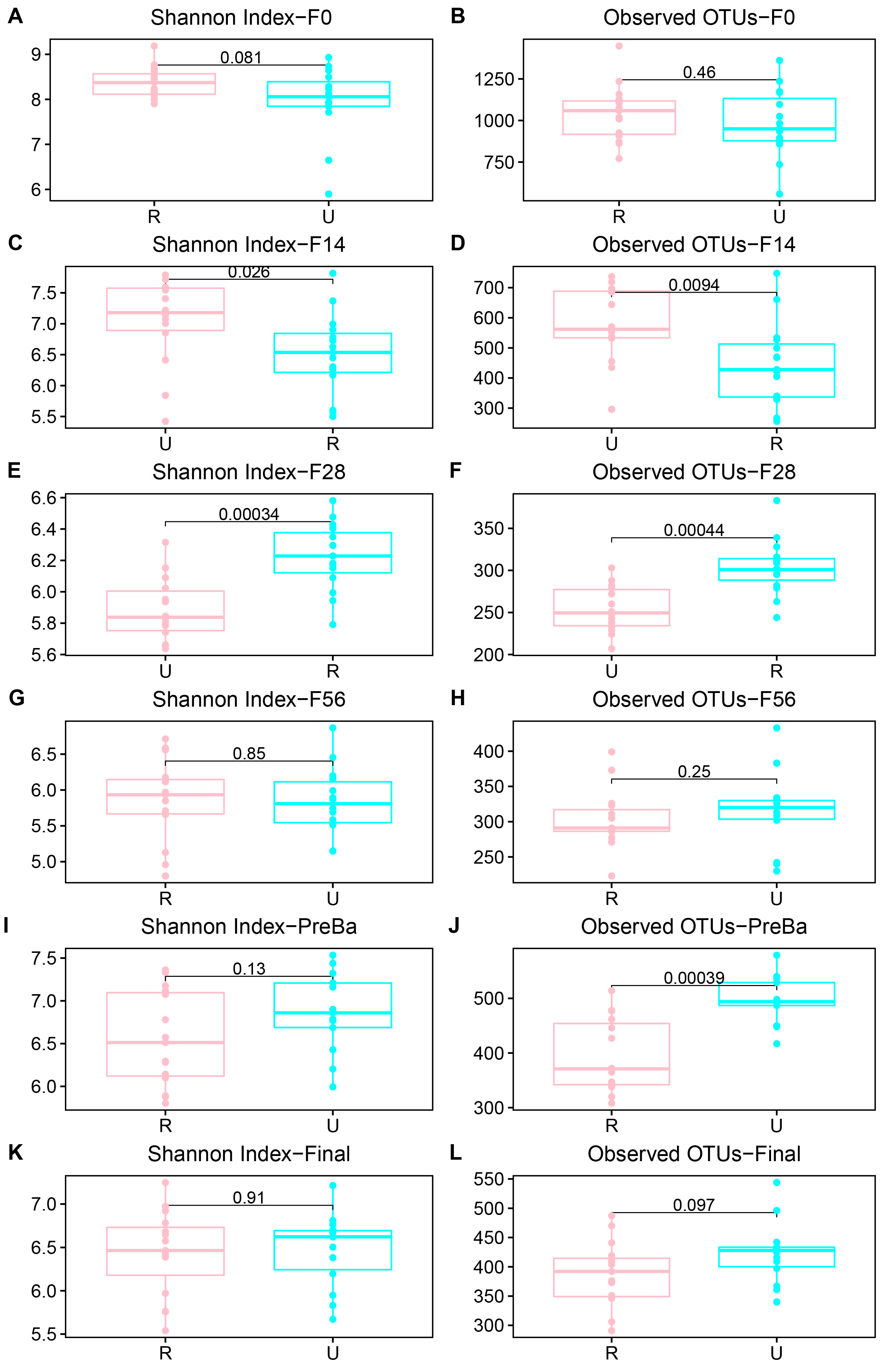


**Fig. S9** Effect of monensin on the alpha diversity in the rumen during the finishing phase. R: cattle consumed monensin; U: cattle did not consume monensin


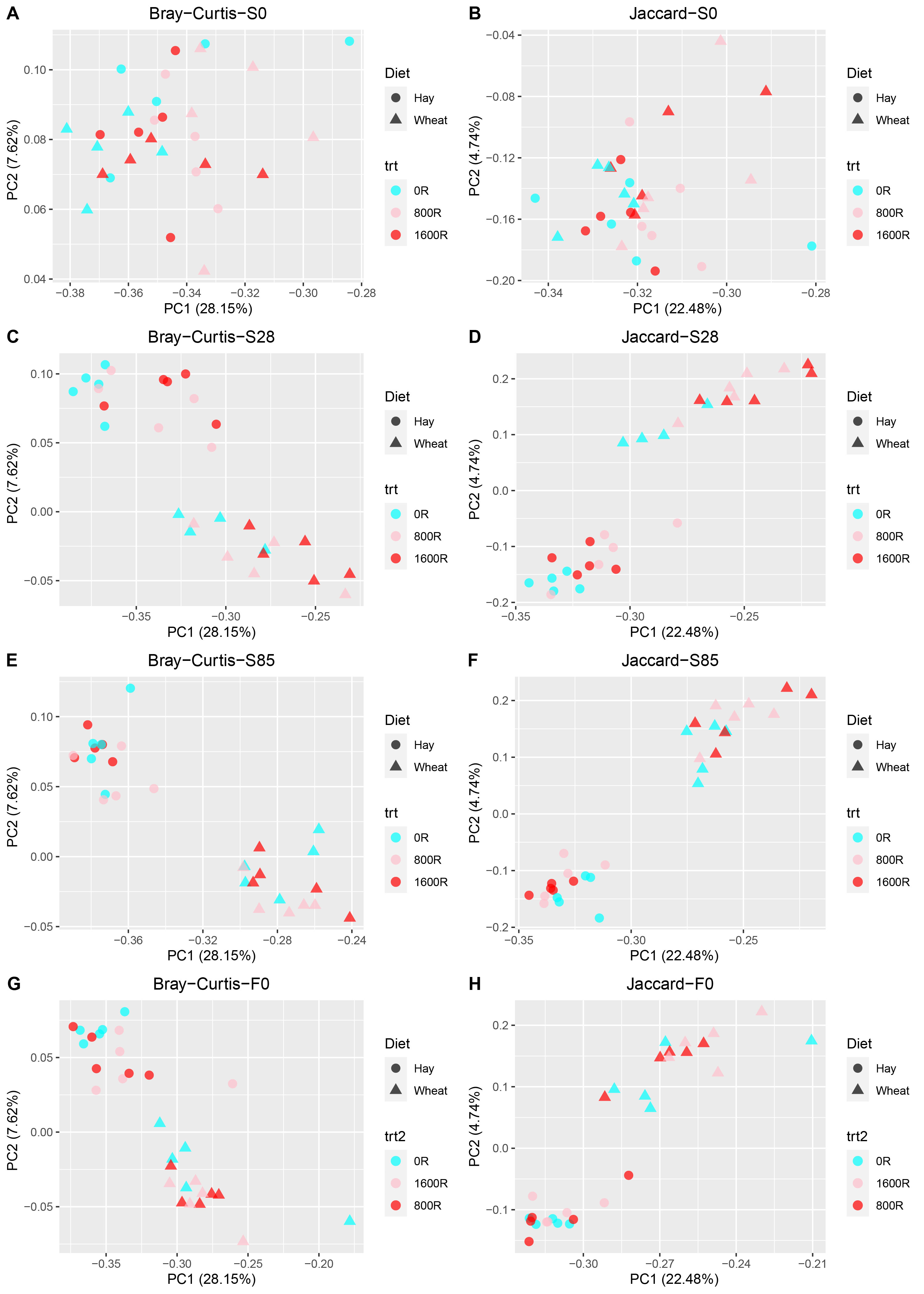


**Fig. S10** Effect of diet and monensin on beta diversity in the rumen during the stocker phase. R: cattle consumed monensin; U: cattle did not consume monensin


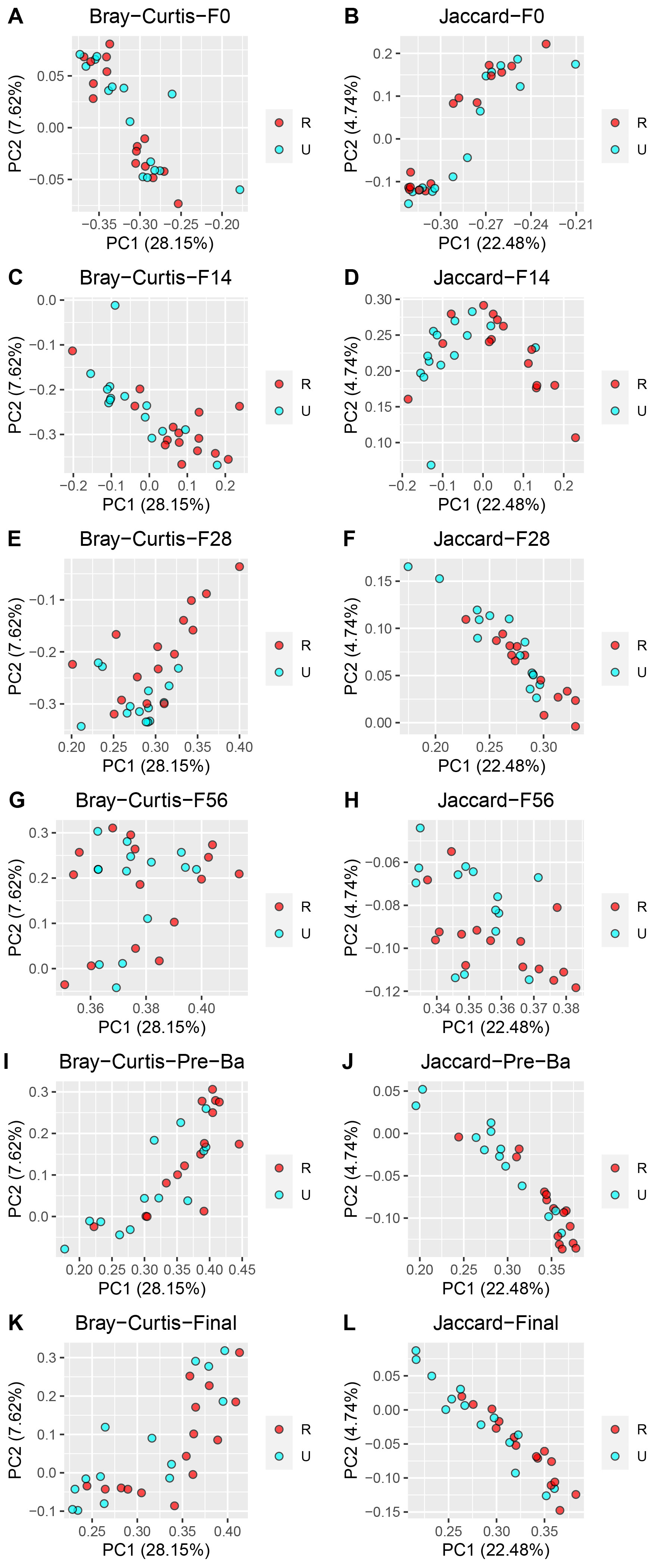


**Fig. S11** Effect of monensin on beta diversity in the rumen during the finishing phase. R: cattle consumed monensin; U: cattle did not consume monensin


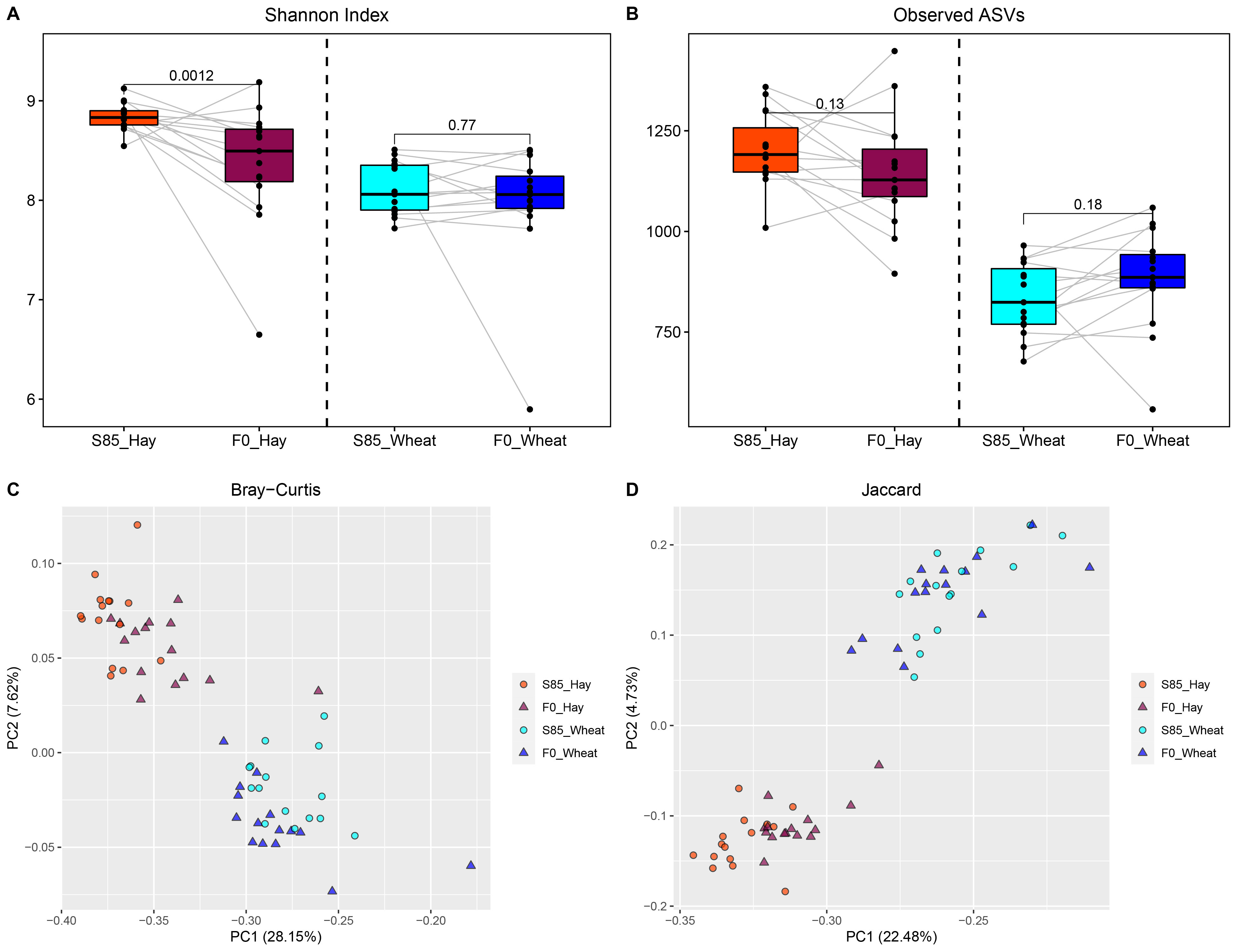


**Fig. S12** Beta diversity in the rumen of cattle consuming hay and wheat diet before and after transportation


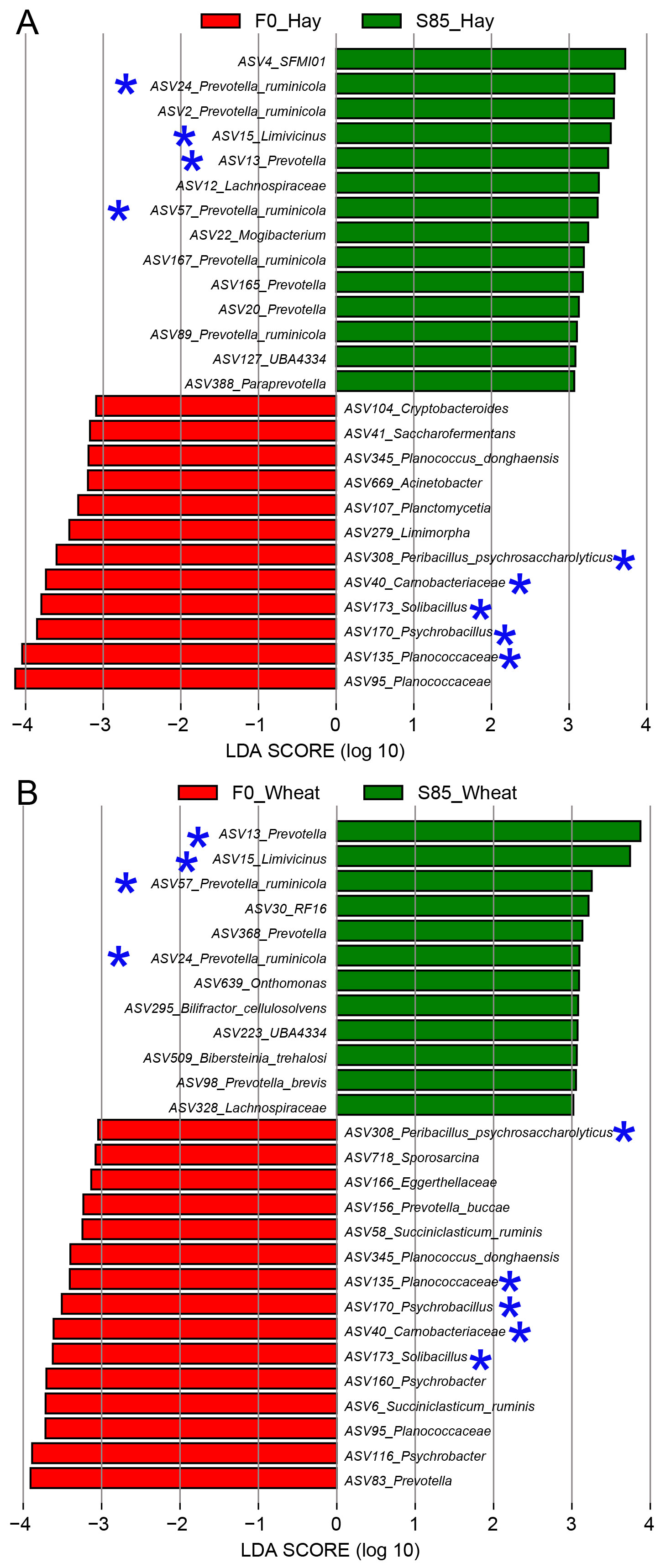


**Fig. S13** Transportation associated bacteria identified by LEfSe


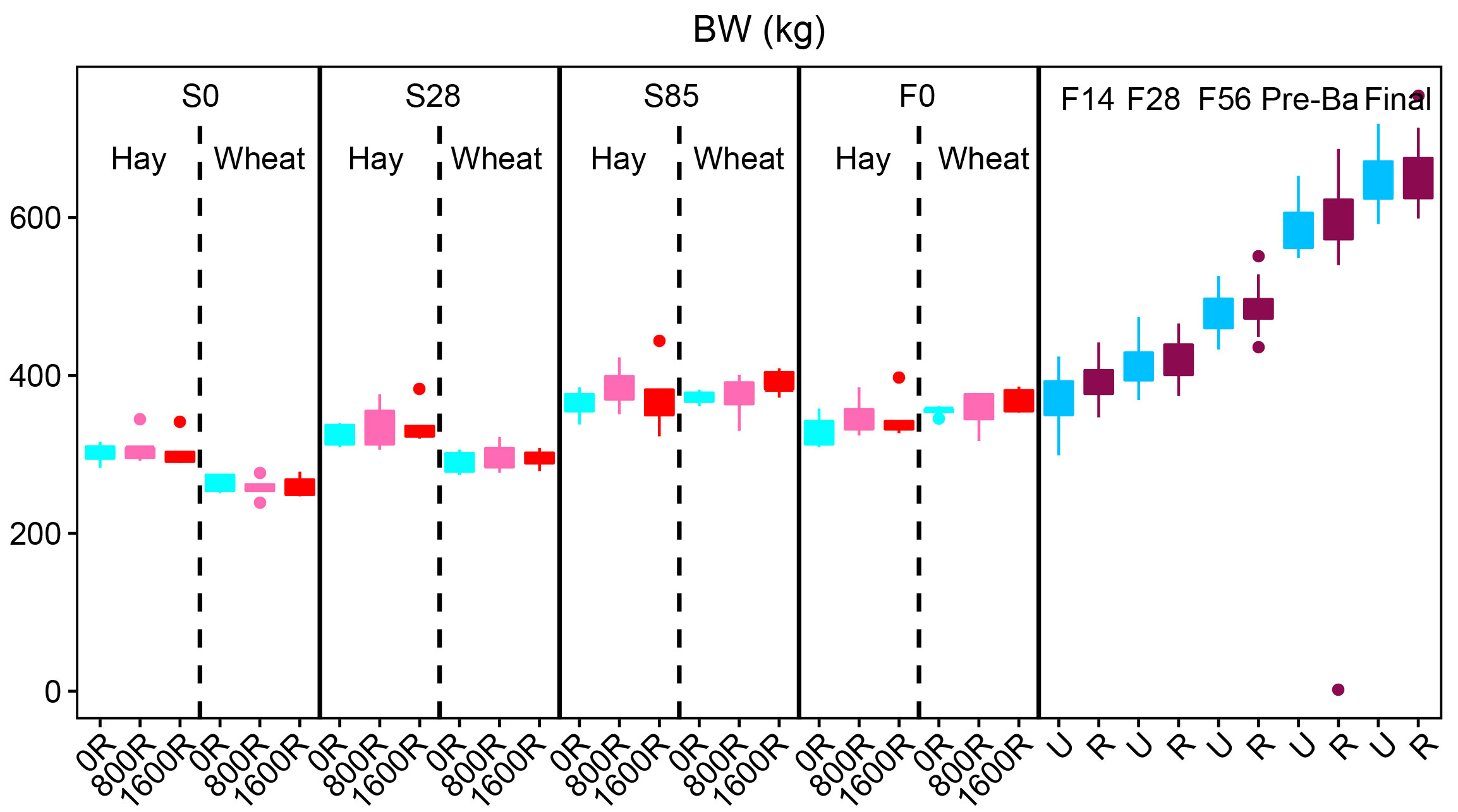


**Fig. S14** Body weight of cattle from the stocker to finishing stage. R=Monensin, U=No monensin. Three levels (0, 800, 1600) of monensin during the stocker stage were designed. Ruminal fluids were collected on day 0, 28, 85 (S0, S28, S85) during the stocker phase and on day 0, 14, 28, 56, starting to add Pre-Ba (30 days before harvest) and at the end of finishing stage (Final) (F0, F14, F28, F56, Pre-Ba, and Final) during the finishing stage

**Table S1** Differences in alpha diversities of the rumen microbiome at different growth stages

| **Shannon Index** | | | **Observed ASVs** | | |
| --- | --- | --- | --- | --- | --- |
| **Group 1** | **Group 2** | ***P*** | **Group 1** | **Group 2** | ***P*** |
| S0 | S28 | 0.000 | S0 | S28 | 0.000 |
| S0 | S85 | 0.000 | S0 | S85 | 0.000 |
| S0 | F0 | 0.000 | S0 | F0 | 0.000 |
| S0 | F14 | 0.000 | S0 | F14 | 0.000 |
| S0 | F28 | 0.000 | S0 | F28 | 0.000 |
| S0 | F56 | 0.000 | S0 | F56 | 0.000 |
| S0 | Pre-Ba | 0.000 | S0 | Pre-Ba | 0.000 |
| S0 | Final | 0.000 | S0 | Final | 0.000 |
| S28 | S85 | 0.474 | S28 | S85 | 0.897 |
| S28 | F0 | 0.007 | S28 | F0 | 0.682 |
| S28 | F14 | 0.000 | S28 | F14 | 0.000 |
| S28 | F28 | 0.000 | S28 | F28 | 0.000 |
| S28 | F56 | 0.000 | S28 | F56 | 0.000 |
| S28 | Pre-Ba | 0.000 | S28 | Pre-Ba | 0.000 |
| S28 | Final | 0.000 | S28 | Final | 0.000 |
| S85 | F0 | 0.034 | S85 | F0 | 0.935 |
| S85 | F14 | 0.000 | S85 | F14 | 0.000 |
| S85 | F28 | 0.000 | S85 | F28 | 0.000 |
| S85 | F56 | 0.000 | S85 | F56 | 0.000 |
| S85 | Pre-Ba | 0.000 | S85 | Pre-Ba | 0.000 |
| S85 | Final | 0.000 | S85 | Final | 0.000 |
| F0 | F14 | 0.000 | F0 | F14 | 0.000 |
| F0 | F28 | 0.000 | F0 | F28 | 0.000 |
| F0 | F56 | 0.000 | F0 | F56 | 0.000 |
| F0 | Pre-Ba | 0.000 | F0 | Pre-Ba | 0.000 |
| F0 | Final | 0.000 | F0 | Final | 0.000 |
| F14 | F28 | 0.000 | F14 | F28 | 0.000 |
| F14 | F56 | 0.000 | F14 | F56 | 0.000 |
| F14 | Pre-Ba | 0.542 | F14 | Pre-Ba | 0.069 |
| F14 | Final | 0.050 | F14 | Final | 0.003 |
| F28 | F56 | 0.060 | F28 | F56 | 0.011 |
| F28 | Pre-Ba | 0.000 | F28 | Pre-Ba | 0.000 |
| F28 | Final | 0.000 | F28 | Final | 0.000 |
| F56 | Pre-Ba | 0.000 | F56 | Pre-Ba | 0.000 |
| F56 | Final | 0.000 | F56 | Final | 0.000 |
| Pre-Ba | Final | 0.072 | Pre-Ba | Final | 0.047 |

**Table S2** Dissimilarities in the rumen microbiome at different growth stages revealed by analysis of similarity (ANOSIM) based on Bray-Curtis distances

| **Group 1** | **Group 2** | ***r*** | ***P* value** |
| --- | --- | --- | --- |
| F0 | F14 | 0.87 | 0.001 |
| F0 | F28 | 1.00 | 0.001 |
| F0 | F56 | 1.00 | 0.001 |
| F0 | Final | 1.00 | 0.001 |
| F0 | Pre-Ba | 1.00 | 0.001 |
| F0 | S0 | 0.65 | 0.001 |
| F0 | S28 | 0.38 | 0.001 |
| F0 | S85 | 0.21 | 0.001 |
| F14 | F28 | 0.73 | 0.001 |
| F14 | F56 | 0.94 | 0.001 |
| F14 | Final | 0.90 | 0.001 |
| F14 | Pre-Ba | 0.89 | 0.001 |
| F14 | S0 | 0.88 | 0.001 |
| F14 | S28 | 0.85 | 0.001 |
| F14 | S85 | 0.86 | 0.001 |
| F28 | F56 | 0.86 | 0.001 |
| F28 | Final | 0.82 | 0.001 |
| F28 | Pre-Ba | 0.84 | 0.001 |
| F28 | S0 | 1.00 | 0.001 |
| F28 | S28 | 1.00 | 0.001 |
| F28 | S85 | 1.00 | 0.001 |
| F56 | Final | 0.43 | 0.001 |
| F56 | Pre-Ba | 0.28 | 0.001 |
| F56 | S0 | 1.00 | 0.001 |
| F56 | S28 | 1.00 | 0.001 |
| F56 | S85 | 1.00 | 0.001 |
| Final | Pre-Ba | 0.08 | 0.011 |
| Final | S0 | 1.00 | 0.001 |
| Final | S28 | 1.00 | 0.001 |
| Final | S85 | 1.00 | 0.001 |
| Pre-Ba | S0 | 1.00 | 0.001 |
| Pre-Ba | S28 | 1.00 | 0.001 |
| Pre-Ba | S85 | 1.00 | 0.001 |
| S0 | S28 | 0.30 | 0.001 |
| S0 | S85 | 0.42 | 0.001 |
| S28 | S85 | 0.10 | 0.004 |

**Table S3** Dissimilarities in the rumen microbiome at different growth stages revealed by analysis of similarity (ANOSIM) based on Jaccard distances

| **Group 1** | **Group 2** | ***r*** | ***P* value** |
| --- | --- | --- | --- |
| F0 | F14 | 0.88 | 0.001 |
| F0 | F28 | 1.00 | 0.001 |
| F0 | F56 | 1.00 | 0.001 |
| F0 | Final | 1.00 | 0.001 |
| F0 | Pre-Ba | 1.00 | 0.001 |
| F0 | S0 | 0.64 | 0.001 |
| F0 | S28 | 0.29 | 0.001 |
| F0 | S85 | 0.21 | 0.001 |
| F14 | F28 | 0.82 | 0.001 |
| F14 | F56 | 0.93 | 0.001 |
| F14 | Final | 0.92 | 0.001 |
| F14 | Pre-Ba | 0.91 | 0.001 |
| F14 | S0 | 0.93 | 0.001 |
| F14 | S28 | 0.83 | 0.001 |
| F14 | S85 | 0.87 | 0.001 |
| F28 | F56 | 0.77 | 0.001 |
| F28 | Final | 0.92 | 0.001 |
| F28 | Pre-Ba | 0.89 | 0.001 |
| F28 | S0 | 1.00 | 0.001 |
| F28 | S28 | 1.00 | 0.001 |
| F28 | S85 | 1.00 | 0.001 |
| F56 | Final | 0.61 | 0.001 |
| F56 | Pre-Ba | 0.46 | 0.001 |
| F56 | S0 | 1.00 | 0.001 |
| F56 | S28 | 1.00 | 0.001 |
| F56 | S85 | 1.00 | 0.001 |
| Final | Pre-Ba | 0.10 | 0.008 |
| Final | S0 | 1.00 | 0.001 |
| Final | S28 | 1.00 | 0.001 |
| Final | S85 | 1.00 | 0.001 |
| Pre-Ba | S0 | 1.00 | 0.001 |
| Pre-Ba | S28 | 1.00 | 0.001 |
| Pre-Ba | S85 | 1.00 | 0.001 |
| S0 | S28 | 0.35 | 0.001 |
| S0 | S85 | 0.41 | 0.001 |
| S28 | S85 | 0.08 | 0.022 |

**Table S4** Dissimilarities in the rumen microbiome of cattle consuming different diets revealed by analysis of similarity (ANOSIM) based on Bray-Curtis distance

| **Group 1** | **Group 2** | ***r*** | ***P* value** |
| --- | --- | --- | --- |
| F0_Hay | F0_Wheat | 0.79 | 0.001 |
| F0_Hay | S0_Hay | 0.88 | 0.001 |
| F0_Hay | S0_Wheat | 0.79 | 0.001 |
| F0_Hay | S28_Hay | 0.78 | 0.001 |
| F0_Hay | S28_Wheat | 0.97 | 0.001 |
| F0_Hay | S85_Hay | 0.57 | 0.001 |
| F0_Hay | S85_Wheat | 0.98 | 0.001 |
| F0_Wheat | S0_Hay | 0.95 | 0.001 |
| F0_Wheat | S0_Wheat | 0.94 | 0.001 |
| F0_Wheat | S28_Hay | 0.95 | 0.001 |
| F0_Wheat | S28_Wheat | 0.68 | 0.001 |
| F0_Wheat | S85_Hay | 0.88 | 0.001 |
| F0_Wheat | S85_Wheat | 0.34 | 0.001 |
| S0_Hay | S0_Wheat | 0.01 | 0.349 |
| S0_Hay | S28_Hay | 0.32 | 0.001 |
| S0_Hay | S28_Wheat | 0.97 | 0.001 |
| S0_Hay | S85_Hay | 0.73 | 0.001 |
| S0_Hay | S85_Wheat | 0.97 | 0.001 |
| S0_Wheat | S28_Hay | 0.28 | 0.001 |
| S0_Wheat | S28_Wheat | 0.96 | 0.001 |
| S0_Wheat | S85_Hay | 0.50 | 0.001 |
| S0_Wheat | S85_Wheat | 0.96 | 0.001 |
| S28_Hay | S28_Wheat | 0.95 | 0.001 |
| S28_Hay | S85_Hay | 0.41 | 0.001 |
| S28_Hay | S85_Wheat | 0.96 | 0.001 |
| S28_Wheat | S85_Hay | 0.87 | 0.001 |
| S28_Wheat | S85_Wheat | 0.42 | 0.001 |
| S85_Hay | S85_Wheat | 0.90 | 0.001 |

**Table S5** Dissimilarities in the rumen microbiome of cattle consuming different diets revealed by analysis of similarity (ANOSIM) based on Jaccard distance

| **Group 1** | **Group 2** | ***r*** | ***P* value** |
| --- | --- | --- | --- |
| F0_Hay | F0_Wheat | 0.99 | 0.001 |
| F0_Hay | S0_Hay | 0.96 | 0.001 |
| F0_Hay | S0_Wheat | 0.90 | 0.001 |
| F0_Hay | S28_Hay | 0.85 | 0.001 |
| F0_Hay | S28_Wheat | 1.00 | 0.001 |
| F0_Hay | S85_Hay | 0.63 | 0.001 |
| F0_Hay | S85_Wheat | 1.00 | 0.001 |
| F0_Wheat | S0_Hay | 1.00 | 0.001 |
| F0_Wheat | S0_Wheat | 1.00 | 0.001 |
| F0_Wheat | S28_Hay | 1.00 | 0.001 |
| F0_Wheat | S28_Wheat | 0.68 | 0.001 |
| F0_Wheat | S85_Hay | 1.00 | 0.001 |
| F0_Wheat | S85_Wheat | 0.45 | 0.001 |
| S0_Hay | S0_Wheat | 0.08 | 0.031 |
| S0_Hay | S28_Hay | 0.34 | 0.001 |
| S0_Hay | S28_Wheat | 1.00 | 0.001 |
| S0_Hay | S85_Hay | 0.63 | 0.001 |
| S0_Hay | S85_Wheat | 1.00 | 0.001 |
| S0_Wheat | S28_Hay | 0.32 | 0.001 |
| S0_Wheat | S28_Wheat | 1.00 | 0.001 |
| S0_Wheat | S85_Hay | 0.46 | 0.001 |
| S0_Wheat | S85_Wheat | 1.00 | 0.001 |
| S28_Hay | S28_Wheat | 1.00 | 0.001 |
| S28_Hay | S85_Hay | 0.33 | 0.001 |
| S28_Hay | S85_Wheat | 1.00 | 0.001 |
| S28_Wheat | S85_Hay | 1.00 | 0.001 |
| S28_Wheat | S85_Wheat | 0.42 | 0.001 |
| S85_Hay | S85_Wheat | 1.00 | 0.001 |
